# How does globalization affect COVID-19 responses?

**DOI:** 10.1186/s12992-021-00677-5

**Published:** 2021-05-20

**Authors:** Steve J. Bickley, Ho Fai Chan, Ahmed Skali, David Stadelmann, Benno Torgler

**Affiliations:** 1 School of Economics and Finance, Queensland University of Technology, 2 George St, Brisbane, QLD 4000 Australia; 2 Centre for Behavioural Economics, Society and Technology (BEST), 2 George St, Brisbane, QLD 4000 Australia; 3 Department of Global Economics & Management, University of Groningen, Groningen, The Netherlands; 4 University of Bayreuth, Bayreuth, Germany; 5 CREMA – Centre for Research in Economics, Management, and the Arts, Südstrasse 11, CH-8008 Zürich, Switzerland

**Keywords:** Coronavirus, COVID-19, SARS-CoV-2, Globalization, Travel restriction, Border closure, Health screening, Survival analysis

## Abstract

**Background:**

The ongoing COVID-19 pandemic has highlighted the vast differences in approaches to the control and containment of coronavirus across the world and has demonstrated the varied success of such approaches in minimizing the transmission of coronavirus. While previous studies have demonstrated high predictive power of incorporating air travel data and governmental policy responses in global disease transmission modelling, factors influencing the decision to implement travel and border restriction policies have attracted relatively less attention. This paper examines the role of globalization on the pace of adoption of international travel-related non-pharmaceutical interventions (NPIs) during the coronavirus pandemic. This study aims to offer advice on how to improve the global planning, preparation, and coordination of actions and policy responses during future infectious disease outbreaks with empirical evidence.

**Methods and data:**

We analyzed data on international travel restrictions in response to COVID-19 of 185 countries from January to October 2020. We applied time-to-event analysis to examine the relationship between globalization and the timing of travel restrictions implementation.

**Results:**

The results of our survival analysis suggest that, in general, more globalized countries, accounting for the country-specific timing of the virus outbreak and other factors, are more likely to adopt international travel restrictions policies. However, countries with high government effectiveness and globalization were more cautious in implementing travel restrictions, particularly if through formal political and trade policy integration. This finding is supported by a placebo analysis of domestic NPIs, where such a relationship is absent. Additionally, we find that globalized countries with high state capacity are more likely to have higher numbers of confirmed cases by the time a first restriction policy measure was taken.

**Conclusions:**

The findings highlight the dynamic relationship between globalization and protectionism when governments respond to significant global events such as a public health crisis. We suggest that the observed caution of policy implementation by countries with high government efficiency and globalization is a by-product of commitment to existing trade agreements, a greater desire to ‘learn from others’ and also perhaps of ‘confidence’ in a government’s ability to deal with a pandemic through its health system and state capacity. Our results suggest further research is warranted to explore whether global infectious disease forecasting could be improved by including the globalization index and in particular, the *de jure* economic and political, and de facto social dimensions of globalization, while accounting for the mediating role of government effectiveness. By acting as proxies for a countries’ likelihood and speed of implementation for international travel restriction policies, such measures may predict the likely time delays in disease emergence and transmission across national borders.

**Supplementary Information:**

The online version contains supplementary material available at 10.1186/s12992-021-00677-5.

## Background

The level of complexity around containing emerging and re-emerging infectious diseases has increased with the ease and increased incidence of global travel [1], along with greater global social, economic, and political integration [2]. In reference to influenza pandemics, but nonetheless applicable to many communicable and vector-borne diseases, the only certainty is in the growing unpredictability of pandemic-potential infectious disease emergence, origins, characteristics, and the biological pathways through which they propagate [3]. Globalization in trade, increased population mobility, and international travel are seen as some of the main human influences on the emergence, re-emergence, and transmission of infectious diseases in the twenty-first Century [4, 5].

Emerging and re-emerging infectious diseases have presented major challenges for human health in ancient and modern societies alike [6–10]. The relative rise in infectious disease mortality and shifting patterns of disease emergence, re-emergence, and transmission in the current era has been attributed to increased global connectedness, among other factors [11]. More globalized countries – and, in particular, global cities – are at the heart of human influence on infectious diseases; these modern, densely populated urban centers are highly interconnected with the world economy in terms of social mobility, trade, and international travel [12, 13]. One might assume that given their high susceptibility to infectious diseases, globalized countries would be more willing than less globalized countries to adopt screening, quarantine, travel restriction, and border control measures during times of mass disease outbreaks. However, given their globalized nature, globalized countries are also likely to favor less protectionist policies in general, thus, contradicting the assumption above, perhaps suggesting that counteracting forces are at play: greater social globalization may require faster policy adoption to limit potential virus import and spread through more socially connected populations [14, 15]; greater economic globalization may indicate slower policy adoption due to legally binding travel and trade agreements/regulations, economic losses, and social issues due to family relations that cross borders [16–21]. Greater political globalization may indicate greater willingness to learn from others and/or maintain democratic processes of decision making in global coordination efforts, either way potentially delaying the implementation of travel restrictions. Travel restrictions may also have minimal impact in urban centers with dense populations and travel networks [22]. Moreover, the costs of closing are comparatively higher for open countries than for already protective nations. For example, more globalized countries are more likely to incur financial or economic penalties (e.g., see [23, 24]) when implementing health policies which aim to improve the health of local populations such as import restrictions or bans on certain food groups/products and product labelling. Globalization, after all, is known to promote growth and does so via a combination of three main globalization dimensions: economic integration (i.e., flow of goods, capital and services, economic information, and market perceptions), social integration (i.e., proliferation of ideas, information, culture, and people), and political integration (i.e., diffusion of governance and participation in international coordination efforts) [25, 26]. See Table 1 for examples of data used in the estimation of each (sub)dimension of the KOF globalization index we use in this study.

**Table 1:** Dimensions and sub-components of Globalization^a^

***Dimension***	***Sub-Component***	Examples of Data Points
Economic	Trade	Trade in goods and services, trade partner diversity, trade regulations, trade taxes, tariff rates, and number of trade agreements.
	Financial	Foreign direct investment, portfolio investment, international debt, reserves and income payments, investment restrictions, capital account openness, and international investment agreements.
Social	Interpersonal	International voice traffic, income transfers, international tourism, international students, migration, telephone subscriptions, freedom to visit, number of international airports.
	Informational	Used internet bandwidth, international patents, high technology exports, television access, and internet access and press freedom.
	Cultural	Trade in cultural goods and personal services, international trademarks, presence of McDonald’s and IKEA stores, gender parity, human capital, and civil liberties.
Political		Number of embassies, involvement in UN peace keeping missions, number of international non-government organizations, number of international inter-governmental organizations, international treaties, and treaty partner diversity.

^a^https://ethz.ch/content/dam/ethz/special-interest/dual/kof-dam/documents/Globalization/2019/KOFGI_2019_variables.pdf

Globalization appears to improve population health outcomes such as infant mortality rate (IMR) and life expectancy (LE) regardless of a country’s level of development (i.e., developed, developing, or underdeveloped) [27, 28]. Links between the dimensions of globalization (i.e., social, political, and economic) and general population health are less clear cut [29]. For less developed countries, the economic dimension of globalization appears to provide the strongest determinant in IMR and LE, whereas for more developed countries, the social aspect of globalization is the strongest factor [27]. This suggests that as a country becomes more economically stable, it then moves towards greater social and political integration into global society; and for less developed countries, increased wealth creation through economic integration potentially delivers the greatest increases in population health. In contrast, for low- to middle-income countries, the social and political dimensions of globalization appear most strongly related to the propensity of women to be overweight [30, 31]. This suggests that for the least developed countries, the adoption of western culture, food habits and lifestyle may be detrimental to adult health if not backed up by social and political progress. Hence, it appears there is no definite relationship between the different aspects of globalization (i.e., social, political, and economic), a country’s level of development, and health outcomes that hold across all health contexts. Regardless, trade policies and more generally, globalization, influence both a nation’s determinants of health and the options and resources available to its health policymakers [32].

The influence of open trade agreements, policies favoring globalization and greater social connectedness on the (delayed) timing of travel restrictions during a pandemic would make logical sense. Globalized countries are more likely to incur financial, economic, and social penalties by implementing restrictive measures that aim to improve population health outcomes (e.g., see [23, 24]) and hence, will be less inclined to do so. Further, countries that rely on international students and tourism and have a high number of expatriates living and working abroad might be even less likely to close their borders or implement travel restrictions to avoid (1) increases in support payments or decreases in tax income during times of unforeseen economic upset, (2) negative backlash from media and in political polls, and (3) tit-for-tat behaviors from major trading partners. However, countries which are more socially connected may also act more quickly because they are inherently at higher risk of local outbreak and hence, to delay local emergence they may implement international travel restrictions earlier. Membership and commitments to international organizations [33], treaties, and binding trade agreements might also prevent or inhibit them from legally doing so [23, 34, 35], suggesting there are social, trade, *and* political motivators to maintain ‘open’ borders.

Domestic policies implemented in response to the coronavirus pandemic have ranged from school closures and public event cancellations to full-scale national lockdowns. Previous research has hinted that democratic countries, particularly those with competitive elections, were quicker to close schools. Interestingly, those with high government effectiveness (i.e., those with high-quality public and civil services, policy formulation, and policy implementation) were slower to implement such policies [36] as were the more right-leaning governments [37]. Further, more democratic countries have tended to be more sensitive to the domestic policy decisions of other countries [38]. In particular, government effectiveness – as a proxy of state capacity – can act as a mediator with evidence available that countries with higher effectiveness took longer to implement COVID-19 related responses [36, 39]. Countries with higher levels of health care confidence also exhibit slower mobility responses among its citizens [40]. Those results may indicate that there is a stronger perception that a well-functioning state is able to cope with such a crisis as a global pandemic like SARS-CoV-2. More globalized countries may therefore take advantage of a better functioning state; weighing advantages and disadvantages of policies and, consequently, slowing down the implementation of restrictive travel policies to benefit longer from international activities. Regardless, the need to understand the reasons (and potential confounding or mediating factors) behind the selection of some policy instruments and not others [36] and the associated timing of such decisions is warranted to enable the development and implementation of more appropriate policy interventions [41].

The literature seems to agree that greater globalization (and the trade agreements and openness which often come with it) make a country more susceptible to the emergence and spread of infectious and noncommunicable diseases [2, 42]. Greater connectedness and integration within a global society naturally increases the interactions between diverse populations and the pathways through which potential pathogens can travel and hence, emerge in a local population. Non-pharmaceutical interventions (e.g., social distancing, city lockdowns, travel restrictions) may serve as control measures when pharmaceutical options (e.g., vaccines) are not yet available [43]. However, such non-pharmaceutical measures are often viewed as restrictive in a social, political, and economic context. Our review of the literature did not detect clear indications of the likelihood that globalized cities will implement such measures, nor were we able to identify how quickly such cities will act to minimize community transmission of infectious diseases and the possible mediating effects of government effectiveness in the decision-making process. Furthermore, our review could not locate research on the relative influence of the social, political, and economic dimensions of globalization on the speed of implementing travel restriction policies. The recent COVID-19 pandemic has highlighted the vast differences in approaches to the control and containment of coronavirus across the world and has demonstrated the varied success of such approaches in minimizing the transmission of coronavirus. Restrictive government policies formerly deemed impossible have been implemented within a matter of months across democratic and autocratic governments alike. This presents a unique opportunity to observe and investigate a plethora of human behavior and decision-making processes. We explore the relative weighting of risks and benefits in globalized countries who balance the economic, social, and political benefits of globalization with a higher risk of coronavirus emergence, spread, and extended exposure. Understanding which factors of globalization (i.e., social, economic, or political) have influenced government public health responses (in the form of travel/border restriction policies) during COVID-19 can help identify useful global coordination mechanisms for future pandemics, and also improve the accuracy of disease modelling and forecasting by incorporation into existing models.

## Methods

### Data

#### Key variables

**Fig. 1:**
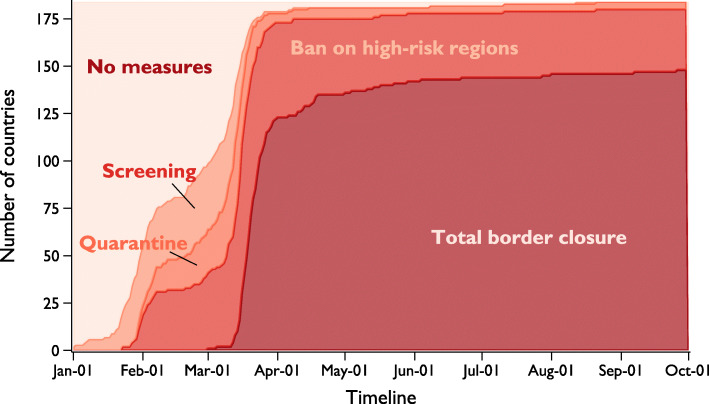
Timeline of international travel restriction policy adoption for 184 countries. Daily count shows the cumulative number of countries that have introduced an international travel policy that is ‘at-least-as-strict’. Relaxation of international travel restriction is not shown in the figure

**Fig. 2:**
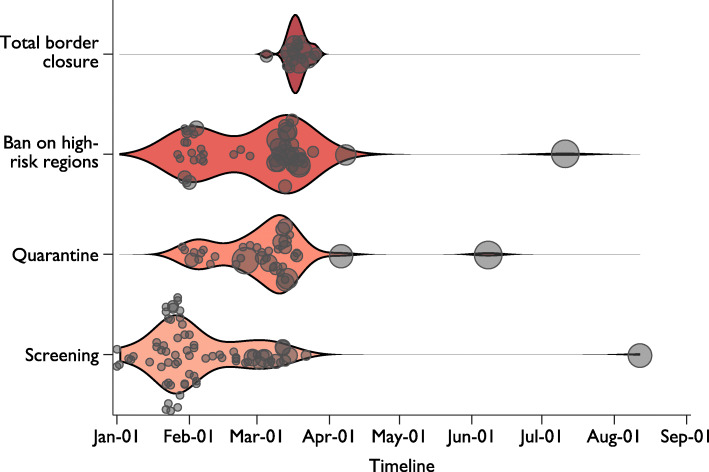
Restrictiveness of the first travel policy implemented over time. Each marker (*N* = 183) represents the type and date of the first travel restriction adopted, with the size of the marker representing the number of confirmed COVID cases at the time of policy implementation. Violin plot shows the kernel (Gaussian) density of timing of implementation

The record for each country’s international travel policy response to COVID-19 is obtained from the Oxford COVID-19 Government Response Tracker (OxCGRT) database [44] (185 countries in total). The database records the level of strictness on international travel from 01 January 2020 to the present (continually updated), categorized into five levels: 0 - no restrictions; 1 - screening arrivals; 2 - quarantine arrivals from some or all regions; 3 - ban arrivals from some regions; and 4 - ban on all regions or total border closure. At various points in time from the beginning of 2020 to the time of writing (06 October 2020), 102 countries have introduced a policy of screening on arrival, 112 have introduced arrival quarantine, 152 have introduced travel bans, and 148 have introduced total border closures.^1^ A visual representation of these statistics in Fig. 1 shows the cumulative daily count of countries that have adopted a travel restriction, according to the level of stringency, between 01 January and 01 October 2020. Countries with a more restrictive policy (e.g., total border closure) and countries with less restrictive policies (e.g., ban on high-risk regions) are also counted. Figure 2 then shows the type of travel restriction and the date each country *first implemented* that policy. Together, we see that countries adopted the first three levels of travel restrictions in two clusters; first between late January to early February, and second during mid-March, around the time that COVID-19 was declared a pandemic by the WHO. Total border closures, on the other hand, were mainly imposed after the pandemic declaration, except for two countries that went into lockdown at the beginning of March (i.e., State of Palestine, and San Marino). Country-specific timelines are shown in Fig. S1 in the Appendix.

We obtained COVID-19 statistics from the European Centre for Disease Prevention and Control (ECDC) and the COVID-19 Data Repository by the Center for Systems Science and Engineering (CSSE) at Johns Hopkins University [45]. The dataset consists of records on the number of confirmed cases and deaths daily for 215 countries since January 2020.

Our measure of globalization is generated from the KOF Globalization Index (of more than 200 countries for the year 2017), published by the KOF Swiss Economic Institute^2^ [26]. The KOF Globalization Index is made up of 44 individual variables (24 de facto and 20 *de jure* components) relating to globalization across economic, social, and political factors^3^^,^^4^ (see also [25]). The complete index is calculated as the average of the de facto and the *de jure* globalization indices. We focus this analysis on the overall index, as well as the subdimensions of globalization (i.e., Economic (Trade and Financial), Social (Interpersonal, Informational, and Cultural), and Political globalization). Additionally, we also investigate the relative contributions of the de facto and *de jure* indices separately. Each index ranges from 1 to 100 (highest globalization). In the regression models, we standardize the variable to mean of zero with unit variance for effect size comparison.

Countries with no records of travel restriction adoption (not included in the Oxford COVID-19 Government Response Tracker) and globalization data from the KOF Globalization Index are listed in Tables S1 and S2, respectively.^5^

#### Control variables

When analyzing the timing of international travel restrictions, we take into account how such decisions can be affected by the policies of neighbors [37, 38]. Thus, to control for policy diffusion, we constructed a variable to reflect international travel policy adoption of neighboring countries by averaging the strictness of each country’s neighbors weighted by the share of international tourism. Inbound tourism data of 197 countries were obtained from the Yearbook of Tourism Statistics of the World Tourism Organization [46]. The data consist of total arrivals of non-resident tourists or visitors at national borders, in hotels, or other types of accommodations; and the overnight stays of tourists, broken down by nationality or country of residence, from 1995 to 2018. Due to differences in statistical availability for each country, we take records from 2018 (or 2017 if 2018 is not available) of arrivals of non-resident tourists/visitors at national borders as the country weights for the computation of foreign international travel policy. If arrival records at national borders are not available for these years, we check for the 2018 or 2017 records on arrivals or overnight stays in hotels or other types of accommodation before relying on records from earlier years. To determine the weighted foreign international restriction policy for each country, we calculated the weighted sum using the share of arrivals of other countries multiplied by the corresponding policy value ranging from 0 to 4.^6^

Similarly, case severity amongst countries comprising the majority of inbound tourists should also increase the likelihood of a country adopting travel restrictions. We thus constructed a variable which takes the sum of the number of confirmed cases from neighboring countries weighted by their share of total arrivals in the focal country (log).

While [47] suggests that the diffusion of social policies is highly linked to economic interdependencies between countries, and is less based on cultural or geographical proximity, we test the sensitivity of our results using a variety of measures of country closeness (Fig. S4 and S5). Doing so also allows us to examine which factors are more likely to predict COVID-19 policy diffusion. In general, while our results are not sensitive to other dimensions of country proximity, decisions to adopt travel restrictions are best explained by models where neighbors are defined by tourism statistics (see *SI Appendix*).

Previous studies have found that countries with higher government effectiveness took longer to implement domestic COVID-19 related policy responses such as school closure (e.g., [36, 39], perhaps due to (mis)perception that a well-functioning state should be able to cope with such a crisis as the current coronavirus pandemic and therefore, has more time or propensity to learn from others and develop well-considered COVID-19 response plans. Therefore, we also control for governance capacity; the data for which is based on measures of state capacity in the *Government Effectiveness* dimension of the 2019 Worldwide Governance Indicators (the World Bank).

We check the robustness of our results using alternative measures such as the International Country Risk Guide (ICRG) *Quality of Government* and tax capacity (tax revenue as % of GDP obtained from the World Development Indicators) [38, 47]. The ICRG measure on the quality of government is computed as the average value of the “Corruption”, “Law and Order”, and “Bureaucracy Quality” indicators. We include additional control variables to account for each country’s macroeconomic conditions, social, political, and geographical characteristics. For macroeconomic conditions, we obtained the latest record of GDP per capita, unemployment rate, and Gini coefficient from the WDI. We include population density, percentage urban population, and share of the population over 65, to control for the social structure of the country, which might affect the odds of implementing the policy due to a higher risk of rapid viral transmission and high mortality rates [38]. We also control for the number of hospital beds in the population [36, 38–40], which we used to proxy for a country’s health system capacity, as countries with higher health capacity may be less likely to implement restrictive travel measures.^7^ We use the electoral democracy index from V-Dem Institute to control for the type of political regime [36, 38, 40]. Following previous studies, we include a dummy variable for countries with prior experience of managing SARS or MERS [38, 48, 49]; defined as those with more than 50 cases. Lastly, we include continent dummies which would absorb any unobserved regional heterogeneity [36]^8^ and country-specific weekend days, as policy changes might have occurred less often on days when politicians are not generally active or at their workplace.

### Empirical strategy

We explore the following questions: how will more globalized countries respond to COVID-19? Do they have more confirmed cases before they first implement travel restrictions? Do they take longer to implement travel restriction policies in general? Which dimension of globalization (i.e., social, political, or economic) contributes most to these responses? To provide answers to these questions, we first report the correlations between the level of globalization and the time gap between the first confirmed domestic case and the implementation date of the first international travel restriction policy, calculated using records from the Oxford COVID-19 Government Response Tracker (OxCGRT [44];) on the timing of restrictions on international travel for each country and COVID-19 case statistics from the ECDC and CSSE [45]. We then examine the relationship using survival analysis through a multiple failure-event framework. This approach allows us to examine the underlying factors which affect the implementation of international travel restriction policies across country borders in an attempt to isolate the effect of globalization. It also allows us to use ‘incomplete’ datasets as certain countries may not have implemented any type of policy or may have implemented a strict policy without first implementing a less strict one (i.e., not sequentially implement policies of ‘least strict’ to ‘most strict’). Furthermore, we conjecture that as a consequence from the above, countries with higher levels of globalization may have more confirmed cases by the time the first policy was introduced. Therefore, we also examine the relationship between globalization and the number of confirmed cases (in logs) at the time of policy implementation.

We employ the time-to-event analysis (survival analysis or event history analysis) to examine the role of globalization in the timing of international travel restriction policies. Similar to previous studies [37, 38, 50], we use the marginal risk set model [51] to estimate the expected duration of time (days) until each policy, with increasing strictness, was imposed by each country. Specifically, we model the hazard for implementing *screening*, *quarantine, ban on high-risk regions*, and *total border closure* separately; thus, allowing the possibility that a country may adopt a more restrictive policy early on, as countries are assumed to be simultaneously at risk for all failures (i.e., implementation of any level of policy strictness). Intuitively, as more stringent policies are less likely to be implemented or adopted early (especially if state capacity is high), we stratified the baseline hazards for the four restrictions to allow for differences in policy adoption rate. Yet, when a country adopts a more restrictive travel restriction policy (e.g., *total border closure*) before (or never) implementing the less restrictive ones (e.g., *ban on high-risk regions*), the latter is effectively imposed (at least from an outcome perspective). Thus, we code them as failure on the day the more restrictive policy was implemented.^9^ We also stratify countries by the month of the first confirmed COVID-19 case,^10^ as countries with early transmission of coronavirus have fewer other countries from which they can learn how best to respond to the pandemic [52]. This is important because disproportionally more countries with a higher globalization index contracted the virus early (Fig. S2 in the *SI Appendix*). Additionally, we stratify time observations into before and after pandemic declaration (11 March 2020) [53] as it is likely to significantly increase the likelihood of countries adopting a travel restriction policy (particularly for border closures as seen in Fig. 2) as consensus on the potential severity of the pandemic solidified. Out of all 184 countries in our sample, 3 and 39 did not implement *ban on high-risk regions* and *total border closure*, respectively, before the end of the sample period, and are thus (right) censored (Fig. 1); i.e., nothing is observed or known about that subject and event after this particular time of observation.

We define the time-at-risk for all countries as the start of the sample period (i.e., 01 January 2020)^11^ and estimate the following stratified (semi-parametric) Cox proportional hazards model [37, 38, 50]:

1 $$ {h}_g\left(\mathrm{t},\mathbf{X}\right)={h}_{0g}\left(\mathrm{t}\right)\ast \exp \left(\boldsymbol{\beta} {\mathbf{X}}_{\mathrm{i}}\right) $$

where *h*_g_(t) is the hazard function of strata *g*, representing the four levels of international travel policy strictness: *screening, quarantine* and *ban on high-risk regions*, and *total border closure*, with *h*_0*g*_ as the respective baseline hazard. Because of the stratification approach, we cluster the standard errors at the country level. Tied failures are handled using the Efron method. The extended Cox model in (1) allows us to include static predictor variables – such as the KOF globalization index – and time-varying covariates on neighboring countries’ international travel policy adoption or daily COVID-19 case statistics to examine their effects, *relative to the baseline hazard,* on the timing of policy implementation in the multiple-events data framework.

To study the relationship between COVID-19 case prevalence and the level of globalization at the time of travel restriction [39], we apply ordinary least squares (OLS) regression models to estimate the following model:

2 $$ {Y}_{ij}=\alpha +\beta {Globalisation}_i+{\gamma}_j{\mathbf{X}}_{\boldsymbol{i}}+{\epsilon}_i $$

where *Y*_*ij*_ is the number of cases (log) at the time of the restriction *j* (or a stricter restriction) was implemented. *Globalisation*_*i*_ is the KOF globalization index of country *i* and **X** is a vector of country-specific controls.

## Results

**Fig. 3:**
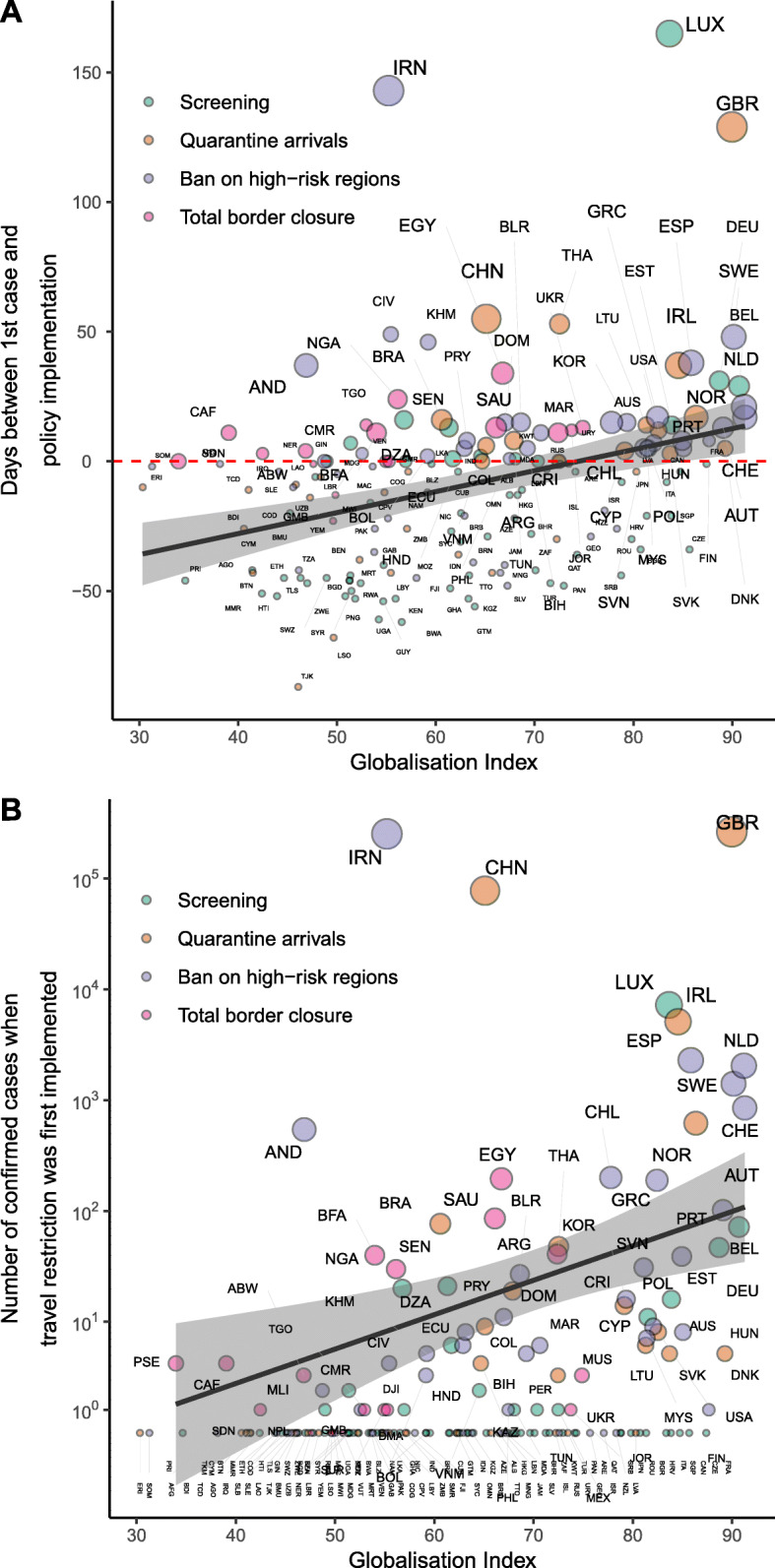
Correlation between the globalization level of a country and **a)** the number of days between the first international travel restriction policy implemented and the first confirmed case; and **b**) the number of confirmed cases (log scale, with countries reporting 0 COVID-19 cases mapped below 1) at the time of the first policy being implemented. The colors represent the four international travel restrictions implemented first in each country. Size of the marker shows the number of confirmed COVID-19 cases on the date of the implementation of the first travel policy

First, we examine whether the level of globalization of the country is correlated with the timing of international travel restrictions relative to the date of a country’s first local confirmed case of coronavirus. With a simple correlation analysis, we find that the Pearson’s correlation between the first policy implementation-first case gap and globalization index is significantly positive *ρ* = 0.35 (*p* < 0.001; 95%CI = [0.210, 0.475]; *n* = 170),^12^ demonstrating that more globalized countries exhibited a delay in imposing travel restrictions compared with less globalized countries (Fig. 3a), relative to their first local confirmed case of COVID-19. Figure 3a also indicates that countries that reacted before the first local COVID-19 case tended to adopt screening on arrivals or quarantine rules as the first precautionary measures. We find that more globalized countries tend to have a higher number of confirmed local cases of COVID-19 at the time of implementing travel restrictions (Pearson’s correlation between the log of confirmed cases and KOF index: *ρ* = 0.408; *p* < 0.001; 95%CI = [0.276, 0.525]; *n* = 173), Fig. 3b).^13^ One noteworthy case is the United Kingdom, which only enforced quarantine on travelers from high-risk regions on the 08 June 2020, 129 days after COVID-19 was first confirmed in the country.

These correlations persist and remain significant when each level of travel restriction is evaluated (see Fig. S3 in *SI Appendix*). This shows that more globalized countries are more likely to impose international travel restrictions later, relative to the first confirmed case in the country, regardless of policy strictness. Interestingly, the two least strict policies (i.e., *screening* and *quarantine*) have slightly higher correlation coefficients meaning that it took more globalized countries longer to impose these policies relative to the first local COVID-19 case. One would think that the least strict policies would represent a lower barrier to continued globalization and hence, be the more likely route for a COVID-19 response measure for more globalized countries.

An intuitive narrative for these findings is that globalized countries are typically among the first to be hit by emerging and re-emerging infectious diseases and are naturally more susceptible to local community transmission [12, 13] (Fig. S2). Hence, globalized countries may have less time to react, strategize, and learn from others in terms of suitable NPIs and how resources need to be mobilized for effective implementation. They may also underestimate the speed of transmission and contagiousness of the virus due to lack of clear evidence and knowledge of the virus at the early stage of the outbreak. Below, we present findings after accounting for the timing of the first COVID-19 wave appearing in the country.

### Do more globalized countries take longer to implement travel restriction policies in general?

We present the results from the survival analysis in Table 2, which shows the hazard ratios (HRs) for each factor. For binary explanatory variables, HRs can be interpreted as the ratio of the likelihood of adopting travel restrictions between the two levels, while for continuous variables, it represents the same ratio for unit difference.

**Table 2:** Time-to-event analysis (marginal risk set model) predicting implementation of international travel restrictions

Model	(1)	(2)	(3)	(4)
KOF Globalization Index	1.08 (0.0739)	1.17^†^ (0.0958)	1.76^**^ (0.363)	1.80^*^ (0.535)
Neighbor restriction adoption		1.30^*^ (0.141)	1.43^***^ (0.146)	1.45^***^ (0.158)
Neighbor COVID-19 case (7-day total, log)		1.09^**^ (0.0312)	1.15^***^ (0.0429)	1.15^***^ (0.0438)
Domestic COVID-19 case (7-day total, log)		1.02 (0.0426)	1.02 (0.0470)	1.02 (0.0493)
Less restrictive travel policy adopted		3.28^***^ (0.577)	3.11^***^ (0.582)	3.14^***^ (0.601)
Weekends		0.49^*^ (0.172)	0.39^**^ (0.133)	0.39^**^ (0.134)
Government Effectiveness (WGI)			0.83 (0.241)	0.83 (0.245)
Electoral democracy index			1.05 (0.109)	1.04 (0.111)
GDP per capita (log)			1.10 (0.193)	1.08 (0.196)
Unemployment (%)			1.04^**^ (0.0152)	1.04^**^ (0.0154)
GINI index			0.99 (0.0104)	0.99 (0.0106)
Hospital beds (per 1 k people)			1.10^*^ (0.0399)	1.09^*^ (0.0403)
Population ages 65+ (%)			0.93^**^ (0.0216)	0.94^**^ (0.0219)
Urban population (%)			1.00 (0.00470)	1.00 (0.00490)
Population density (log)			0.99 (0.0556)	0.99 (0.0563)
MERS or SARS experience			0.78 (0.269)	0.78 (0.270)
Continent				
Africa			0.94 (0.305)	0.95 (0.316)
Asia			1.36 (0.407)	1.38 (0.421)
Central America			0.52 (0.235)	0.54 (0.243)
Europe			(ref.)	(ref.)
North America			1.00 (0.481)	0.99 (0.482)
Oceania			2.32^**^ (0.737)	2.27^*^ (0.726)
South America			1.35 (0.448)	1.40 (0.462)
KOF*Neighbor restriction adoption				0.93 (0.0720)
KOF*Neighbor COVID-19 case (7-day total, log)				1.02 (0.0312)
Num. obs.	55,163	45,484	35,418	35,418
Num. countries	173	158	121	121
Num. failures	655	594	455	455
Pseudo R^2^	0.001	0.037	0.068	0.068
Log likelihood	− 2140.460	− 1813.963	− 1227.150	− 1226.585

Note: Hazard ratios. Standard errors (clustered at country level) in parentheses. † *p* < .10; * *p* < .05; ** *p* < .01; *** *p* < .001

Despite the strong positive correlation observed in the bivariate analysis between globalization and the time difference between first local confirmed case and implementation of travel restriction, we did not find substantial evidence suggesting that more globalized countries are more reluctant to adopt travel restriction policies relative to their first local confirmed case. In fact, after adjusting for the date that COVID-19 was first locally contracted (through observation stratification), we find that, in general, more globalized countries are more likely to adopt travel restriction policies. Specifically, as the KOF globalization index increases by one standard deviation (e.g., from Paraguay to New Zealand), the likelihood of adopting travel restrictions increases by 80% (*p* = 0.007; 95%CI = [1.163, 2.617], Table 2 model 3).

We also find strong evidence of travel restriction policy diffusion between countries that are heavily interdependent in the tourism sector; that is, a country is more likely to adopt a travel restriction if neighboring countries (in terms of share of non-resident visitor arrivals) have done so. As expected, an increase in COVID-19 prevalence in regions comprising the majority of inbound international tourist arrivals increases the likelihood of enforcing travel restrictions. Specifically, for every 1% increase in COVID-19 cases in neighboring countries, the chance of adopting a travel policy increases by about 15% (*p* < 0.001, 95%CI = [1.075, 1.237]). On the other hand, increases in domestic COVID-19 cases do not appear to influence travel policy adoptions^14^ suggesting that travel restriction policy decisions may be driven more by ‘keeping the disease out’ than containing the disease locally for the greater global good. The likelihood of adopting a restrictive travel policy (e.g., arrivals ban) is about three times higher if the country has already implemented a less strict policy, suggesting there may be decreased difficulty in implementing more restrictive policies over time or an increased preference to do so. Moreover, policy change is 60% less likely to occur during weekends (*p* = 0.005, 95%CI = [0.199, 0.757]), perhaps because government officials are less likely to be working on weekends and hence, less active in the political decision-making process.

The effect of the electoral democracy index is not statistically significant, and our results are contrary to the findings of [38], where OECD countries with higher electoral democracy have lower rates of domestic policy adoption.^15^ Perhaps decisions to implement international travel restrictions are less controversial to voters than domestic policies as the former primarily aims at limiting mobility from outside country borders rather than restricting the freedom and mobility within country borders as the latter do. In addition, we find that countries with a higher unemployment rate are more likely to implement travel restrictions. Surprisingly, countries with a larger share of older population are less likely to implement travel restrictions, while no statistically significant effect was observed for the share of urban population and population density. Contrary to our expectation, countries with greater healthcare capacity tend to be more likely to adopt a travel restriction policy.^16^

#### Government capacity as a relevant mediator

**Fig. 4:**
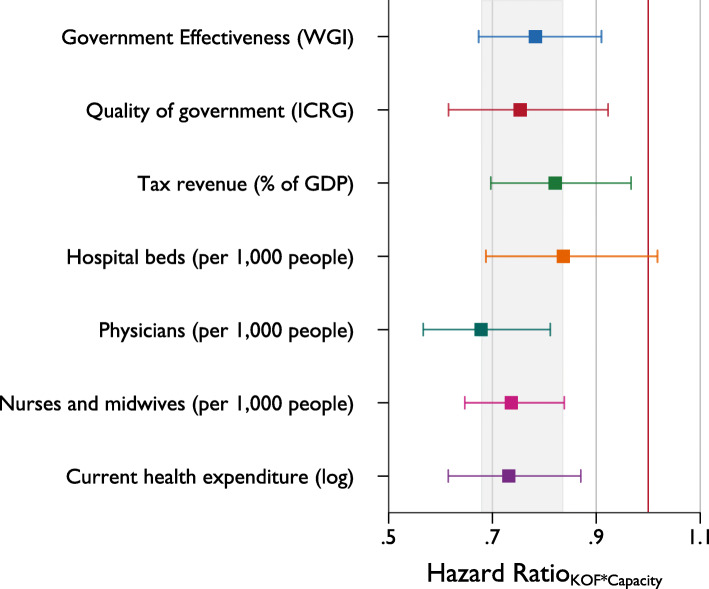
Hazard ratios of interaction terms between globalization and state capacity or health care capacity. Cap represents 95% confidence intervals. Shaded area highlights the range of HRs

When including the interaction term between the globalization index and measures of state capacity in the model, we find strong evidence suggesting that more globalized countries with higher government effectiveness are slower to adopt travel restrictions. On the other hand, the likelihood to adopt travel restrictions increases with the level of globalization for countries with lower state capacity. Perhaps these countries are more self-aware of their lack of preparedness and/or ability to execute effective COVID-19 response plans or accommodate large fluxes of hospital admissions owing to the coronavirus pandemic. Each regression includes the same set of control variables as those used in Table 2 model 4. As shown in Fig. 4, the hazard ratios of the interaction terms between KOF globalization index and WGI government effectiveness are statistically less than one (*p* = 0.001), as well as the interaction term with an alternative measure of state capacity, namely ICRG quality of government (*p* = 0.006) and tax capacity (*p* = 0.018). For instance, computing the hazard ratios of globalization at different levels of government effectiveness reveals that the change in the likelihood to impose travel restrictions, with respect to a one standard deviation increase in KOF, is about 1.5 times higher (hazard ratio of 2.5) for a country with a WGI of 1.5 standard deviations below the world’s average (e.g., Chad) while the risk a country with a WGI 1.5 standard deviations above the world’s average (e.g., Austria) would fell by 12% (hazard ratio of 0.88). Moreover, we also find a similar effect with the interaction terms between globalization and health capacity (as measured by number of hospital beds (*p =* 0.075), physicians (*p* < 0.001), or nurses and midwives per 1000 (*p* < 0.001), and current expenditure on heath (log) (*p* < 0.001)). This evidence supports the notion that countries with higher state or healthcare capacity and globalization were less likely to limit international travel, even when the stakes might be comparatively higher, i.e. when the country is more globalized and hence, more susceptible to infectious disease outbreaks.

### Which aspect of globalization can primarily account for these responses?

**Fig. 5:**
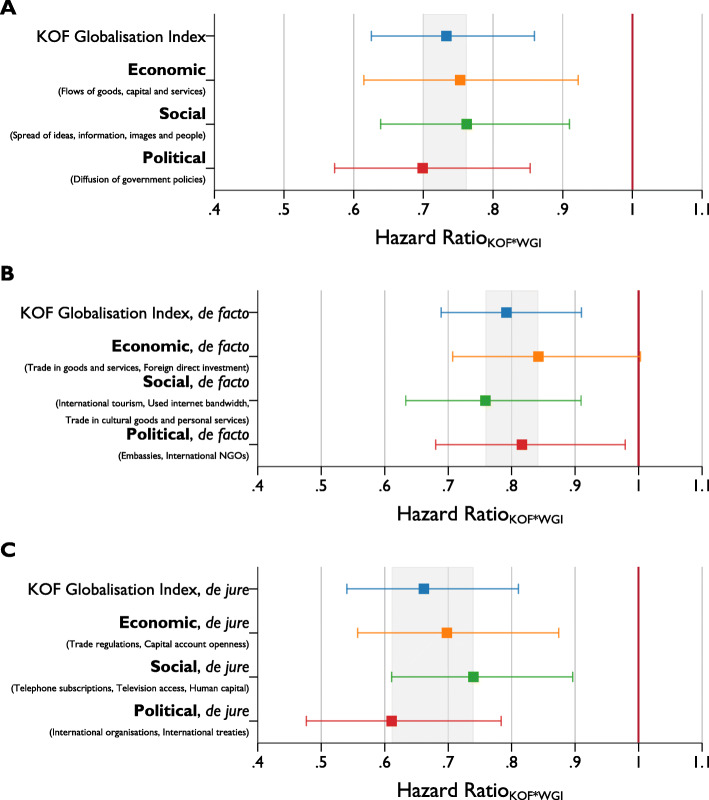
HRs of the interaction terms between government effectiveness and different dimensions of the globalization index on adoption of travel restrictions. Cap represents 95% confidence intervals

Next, we assess which aspects of globalization are more important when predicting travel restriction policy adoption by examining the influence of each (sub)dimension of the globalization index. We find the positive effect of globalization on the likelihood to adopt international travel restrictions is likely to be driven by the social dimension of globalization (Fig. S6, HR is larger than 1 for both *de jure* and de facto dimensions), as the estimates of HR are statistically significant when we re-estimate model 4 in Table 2 with the three subdimensions of KOF. Only the subdimension of social globalization is statistically significant which shows that countries with higher social globalization are quicker to adopt travel restrictions, controlling for other factors. Moreover, we estimate and compare the hazard ratios of the interaction term of each globalization dimension with government effectiveness to assess mediator effects (Fig. 5).^17^ Overall, we find the likelihood of implementing travel restriction policies among countries with high state capacity is robustly estimated for all subcomponents (Fig. 5a), with HRs ranging from 0.70 (political globalization) to 0.76 (social globalization). A closer inspection distinguishing between de facto (actual flows and activities, Fig. 5b) and *de jure* (policies, resources, conditions and institutions, Fig. 5c) measures [26] leads to interesting insights.

First, we find that *de jure* political (number of treaties and memberships in international organizations) globalization, have the largest effect out of all other sub-dimensions of globalization.^18^ This is a highly surprising result given the call for international cooperation and coordination by many international organizations (e.g., WHO,^19^ World Economic Forum,^20^ United Nations^21^). We find that those countries with high government effectiveness and engagement in international political coordination efforts are less likely to implement travel restriction policies and hence, slower to do so. On the other hand, de facto economic globalization, which measures actual economic activities (such as exchange of and goods and services) over long distances, is not as strongly related to the timing of travel policy adoption for countries with high government effectiveness. De facto social globalization has the largest effect among other de facto globalization dimensions. These results suggest that a nation with high government effectiveness and more global social, interpersonal, and cultural flows is less likely to implement travel restriction policies in pandemic crises and hence, may delay doing so. Countries with higher government effectiveness and policies and conditions that tend to facilitate or favor globalization (e.g., trade policy, political connectedness and engagement in international political cooperation) are also less likely to implement travel restrictions.

### Placebo analysis with domestic COVID-19 responses

To assess whether the observed delay in travel restriction adoption is better explained by globalization and its interplay with state capacity, we conduct a placebo analysis using COVID-19 policy responses that, at least in theory, cannot be explained by the same mechanism. Specifically, we employ the same event history analysis on domestic non-pharmaceutical interventions (NPIs) imposed to mitigate COVID-19 transmission. While previous studies have argued for [48] and found a substantial negative effect of government effectiveness on the timeliness of enacting school closure policies [36] and other NPIs across Europe [39], there is no obvious reason why the delayed responses to implement domestic NPIs would be related to globalization. Thus, we would expect that the interaction term between globalization and government effectiveness to be zero. If our expectation is correct, then we are more comfortable interpreting our previous results as truly reflective of the effect of globalization on travel restrictions, rather than as the effect of globalization on the propensity to implement all types of NPIs.

Data on domestic NPIs adoption are derived from the same source we obtained records on international travel restriction (i.e., the OxCGRT database). Domestic containment and closure policies include closing of schools, workplace, and public transport, restriction on gatherings and internal movement, cancellation of public events, and shelter-in-place order. We follow the approach of [38], who focus only on mandatory nationwide policies adopted.^22^ We again utilize the marginal risk set model in analyzing the timing of adoption of the seven domestic policies, that is, we stratified the seven different policies and their variation in strictness. Similarly, adoption of a stricter version of the policy (e.g., restrictions on gatherings between 11 and 100 people or 10 people or less) implies the adoption of the less strict version.

The results of the placebo analysis are presented in Table S3, showing the hazard ratios of each factor predicting the adoption of any COVID-19-related NPIs. Comparison of the estimates of several key variables to previous studies, while subject to a larger set of countries and more complete time frame, suggests that our modelling approach is reasonable.^23^ Similar to the adoption of international travel restrictions, more globalized countries are quicker to implement domestic NPIs than their less globalized counterparts.

Notably, the estimates of HRs are *larger* in magnitude and with higher statistical significance compared to the set in Table 2 for the case of international travel restrictions. This shows that the *relative* speed of more globalized countries in adopting travel restrictions is *slower* than domestic NPIs, compared to less globalized countries, suggesting the former takes relatively more time to impose international travel restrictions, where one would expect international travel policies to be adopted relatively earlier. Thus, this may show that globalized countries are more reluctant, at least relative to the implementation of domestic interventions, to impose international restrictions. This is perhaps due to that domestic NPIs are relatively easier to actualize in more globalized countries, as legally binding international travel and trade agreements and regulations and the potential for massive economic losses [23, 33–35] would also impede the introduction of international travel restriction policies, relative to domestic NPIs. Secondly, and more importantly, we did not find any statistical evidence suggesting the effect of state capacity varies across countries with different levels of globalization as the interaction effect between KOF and government effectiveness is not significant. This result holds for the alternative measures of state capacity as well as using measures of health system capacity. Finally, we also show that the results of the placebo analysis are not sensitive to the type of domestic policy adopted (see Table S4) nor when different dimensions of globalization were considered, as none of the HRs of their interaction terms is statistically significantly smaller than one.^24^

Nevertheless, while the results from the placebo analysis suggest that the results we see in Table 2 are less likely to arise from, e.g., confounding effects due to other unobserved variables, given the difference in nature of domestic and international NPIs,^25^ we cannot conclusively claim that this is in fact the case. For example, an alternative explanation for why more globalized countries respond relatively faster with domestic policies than do less globalized countries might be found in the fact that most of the domestic policies were implemented at a later stage of the pandemic (compared to travel restrictions which were typically adopted early on). Hence, globalized countries may be better at learning how to coordinate resources and implement social distancing policies.

### COVID-19 case severity at the introduction to international travel restriction policies

We conduct an analysis using the Ordinary Least Squares model predicting the number of confirmed COVID-19 cases when each travel restrictions were implemented.^26^ In each regression, we control for the date when the country has the first confirmed COVID-19 case. For countries with no confirmed cases when the travel restriction was implemented (i.e., date of the first confirmed is later than the date of the policy adoption), we recode this variable to the date when the policy was adopted.

**Fig. 6:**
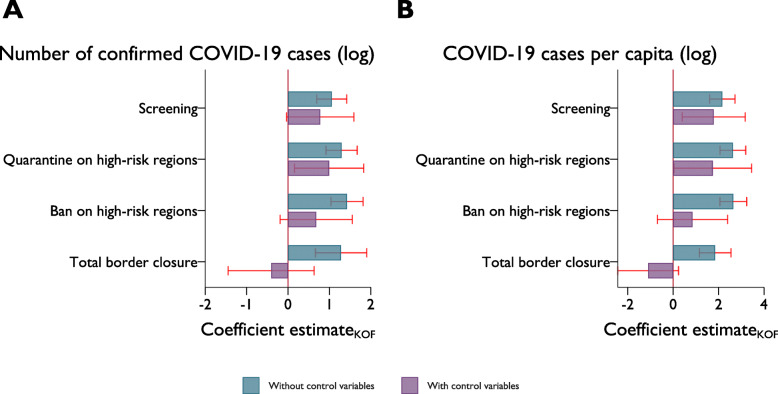
Coefficients of globalization index predicting the number of COVID-19 cases at the time of travel restriction. Cap represents 95% confidence intervals. Full regression results are presented in Table S5 and Table S6

In Fig. 6, we present the estimates of KOF globalization index on COVID-19 prevalence (total number of cases in log (6A) and case per capita in log (6B)) at the time the travel restriction was implemented. We report the estimates obtained from the models without controlling for other factors except for the date of the first confirmed case and models in which we include a full set of control variables (full regression results are presented in Table S5 and Table S6). This includes government effectiveness, electoral democracy, GDP per capita, unemployment rate, GINI coefficient, number of hospital bed per 1000 people, urban population, population density, whether the country experienced SARS or MERS, and region dummies. Additionally, we also control for containment policies implemented before the introduction of the travel restrictions of interest. We proxy this variable by the average value of the stringency index from the beginning of the time period to the day before the travel policy was adopted.^27^

We find strong positive associations between the globalization index and the number of confirmed COVID-19 cases (and per capita cases) at the time the travel restriction policy was first introduced when we only account for when the country was first exposed to COVID-19. In particular, with a one standard deviation increase in globalization index, the predicted number of COVID-19 cases increases by about 1.9 times when screening (or more strict policies) was first adopted, while cases per capita are 7.7 times higher. The globalization multiplier in COVID-19 cases (or cases per capita) is higher when considering firmer travel restrictions (i.e., adoption of quarantine and banning entry from high-risk regions) except for total lockdown. However, the coefficient estimates for globalization predicting COVID-19 cases at the time of total border closure is likely to be underestimated, as a number of highly globalized countries, such as the USA, Japan, South Korea, and a large group of European countries (with the exception of Germany) did not totally close their borders at any point.

Except for the adoption of screening and quarantine, the effect of globalization became statistically insignificant when other control variables are added to the model. The reduction in the effect size is not unexpected as globalization index is highly correlated with several control variables, such as GDP per capita (ρ = 0.631), government effectiveness (ρ = 0.751), and share of the population over 65 (ρ = 0.775).

Additionally, we find further evidence supporting the mediating role of state capacity to the effect of globalization as suggested by the statistically significant interaction effect between globalization and government effectiveness (Table 3). That is, among globalized countries, those with higher state capacity are more likely to have more COVID-19 cases when the government first imposes travel restrictions. This echoes the findings from the time-to-event analysis.

**Table 3:** State capacity mediating effect on globalization

	Screening	Quarantine	Ban high-risk	Total lockdown
KOF Globalization Index	0.92^*^ (0.458)	1.18^**^ (0.415)	0.85^†^ (0.436)	−0.24 (0.487)
Government Effectiveness (WGI)	0.023 (0.377)	−0.25 (0.413)	0.14 (0.467)	0.24 (0.467)
KOF*WGI	0.37^†^ (0.204)	0.47^*^ (0.202)	0.44^*^ (0.196)	0.53^**^ (0.185)
Controls	Yes	Yes	Yes	Yes
Number of countries	118	118	118	118
Prob. > *F*	0.000	0.000	0.000	0.000
*R*^*2*^	0.759	0.718	0.709	0.835

Notes: OLS estimates. Dependent variable: Number of confirmed COVID-19 cases (log) at time of travel policy adoption. Standard errors (heteroskedasticity-robust) in parentheses. † *p* < .10; * *p* < .05; ** *p* < .01; *** *p* < .001

## Discussion

Non-pharmaceutical interventions such as travel restrictions may be seen an immediate means by which governments can delay infectious disease emergence and transmission [43], particularly during the early stages of a pandemic when pharmaceutical interventions such as vaccines are not available [43]. To our knowledge, this study is the first to explore the influence of globalization on the timing of international travel restrictions implemented during the recent coronavirus pandemic and the mediating effect of government effectiveness. From a sample of more than 100 countries, we observe that in general, more globalized countries are more likely to implement international travel restrictions policies than their less globalized counterparts. However, we also find that more globalized countries tend to have a higher number of domestic COVID-19 cases before implementing their first travel restriction and also react slower to their first confirmed domestic case of COVID-19. Additionally, we find that countries with a higher level of globalization may be *relatively* more reluctant to impose international travel restrictions compared to domestic social isolation policies as the effect of globalization on the likelihood to implement the former is smaller than the latter.

Among globalized nations, those with high measures of government effectiveness are less likely to impose international travel restriction policies, suggesting some mediating effect. Perhaps their lower likelihood to implement travel restriction policies is due to (over)confidence in their capability and resources to deal with disease outbreaks, particularly true for some North American and European countries with substantial health system capacity but limited recent experiences with such pandemics [48]. It may also be that high government effectiveness is associated with mechanisms to better evaluate potential costs and benefits of implementing different measures or require approvals, coordination, and action across various levels of (sometimes conflicting) governance. In particular, the interaction variables between government effectiveness and *de jure* political and economic globalization metrics (i.e., representing policies, trade agreements, and pre-conditions which support greater global mobility and trade) have the largest influence on the likelihood to adopt travel restrictions out of all (sub)dimensions of globalization. Perhaps because the penalties from restrictive travel policies are not insignificant, countries with high government effectiveness and more formalized economic and political integration are more inclined to spend time considering the advantages (e.g., delayed domestic COVID-19 emergence) and disadvantages (e.g., reduced trade and potential conflicts with incumbent trade partners) of travel restrictions because the disadvantages affect them so disproportionately. Out of the interactions between government effectiveness and de facto measures, social measures of globalization have the greatest influence on likelihood to implement travel restrictions. Perhaps a nation with high government effectiveness and more global social, interpersonal, and cultural flows needs more time to consider the practicality of implementing travel restrictions or have their hands tied by commitments to international treaties and travel agreements (i.e., they must maintain ‘open’ borders to honor their incumbent commitments) [23, 33–35]. Given this evidence, we propose that interaction variables between government effectiveness and (sub)dimensions of globalization may be suitable proxies in infectious disease models for the likelihood of a country implementing travel and border restriction policies during a global health crisis such as COVID-19.

Countries often implement policies similar to those employed by their major economic partners, rather than those of close cultural or geographical proximity [55]. They also tend to emulate policy interventions of ‘successful’ foreign incumbents [56], suggesting some degree of knowledge or information transfer. However, during the early days of a pandemic, there may be limited ‘successful’ nations to learn from. Our study provides further support to the former proposition: countries are more likely to implement a travel restriction policy if their nearest neighbor (in terms of share of non-resident visitor arrivals) does. The implementation of travel restrictions is related more strongly to confirmed cases in neighbor countries than it is to domestic cases; perhaps this is due to the aim of the policy to keep the disease out rather than minimize spread between nations. Finally, we also find that the likelihood of adopting a more restrictive travel policy (e.g., arrivals ban) is about three times higher if the country has already implemented a less strict policy, suggesting reduced inertia in enacting more restrictive policies once the first measure has been taken.

The benefits of incorporating individual behavioral reactions and governmental policies when modelling the recent coronavirus outbreak in Wuhan, China has been demonstrated [57] and the usefulness of including air travel in the modelling of global infectious disease transmission has been shown [58, 59]. Some empirical evidence points to a small yet significant positive relationship between the implementation of international travel restrictions and the time delay in infectious disease emergence and transmission in the focal country [22, 60, 61]. Broader policy evaluations are still missing. Our results indicate it might be reasonable to assume that global infectious disease forecasting could be improved by including the globalization index while accounting for the mediating role of government effectiveness. In particular, the *de jure* economic and political dimensions and de facto social dimensions could serve as proxies for an effective government’s likelihood and speed to implement travel restriction policies and hence, to predict the likely time delays in disease emergence and transmission across national borders [62]. include domestic, nationwide pandemic policies in their model with results to suggest that such policies are effective and promptly enforced to demonstrate the greatest benefits. While the results from this study might suggest that including international travel restriction policies could bolster additional support for the adoption of such policies in times of mass disease outbreak, it is important to remember that travel restrictions do not (typically) completely mitigate the emergence of infectious diseases, instead delaying the importation of infectious diseases and potentially minimizing the overall severity of outbreak [43, 60] and hence, reducing the associated demand for health system resources at the same time. Geographical regions known hotspots for the emergence and re-emergence of infectious agents [63, 64] could be considered as early candidates for inbound country-specific travel restrictions in the event of mass disease outbreaks.

Due to the ongoing state of COVID-19 transmission and continued enforcement of travel restriction policies, we are not yet able to fully explore the relationship between globalization and the easing of travel restrictions over time. As this data becomes available in the coming months, we will be able to explore various phenomena related to globalization and the easing of international travel restrictions; for example, where nations open up too early (i.e., are these nations overconfident in their health system capability?) or the sequence of easing travel restriction (i.e., do more globalized countries lift restrictions entirely in one go or do they go from strict to less strict?). To this end, further research is required to assess the drivers behind a nation’s decision to (not) close its border in a timely fashion, despite their level of globalization.

In any analysis seeking insights based on government-based data sources, there is concern regarding the availability and quality of reporting as well as the difficulties in drawing robust policy recommendations using these data and the research design of the study. We control for this by incorporating into our analyses a wide and varied set of data sources and analytical tools. In doing so, we aim to strengthen our findings by demonstrating multiple routes/methods to reach similar conclusions. Nevertheless, care should be taken in interpreting the results of our analyses as correlation does not mean causation. However, our findings seem to provide strong support for the notion that, in general, more globalized countries are more likely to implement travel restriction policies. However, if they are also high in government effectiveness, they tend to be more hesitant to implement travel restriction policies (both domestic and international), particularly when high in *de jure* economic and political globalization and de facto social globalization. Thus, suggesting some non-insignificant mediating effect. Additionally, measurement errors stemming from states underreporting of outbreaks due to fear of financial losses or lack of testing capacities [18] could also contribute to the explanations of our results.

## Conclusion

The recent COVID-19 pandemic highlights the vast differences in approaches to the control and containment of infectious diseases across the world, and demonstrates their varying degrees of success in minimizing the transmission of coronavirus. This paper examines the influence of globalization, its (sub)dimensions, and government efficiency on the likelihood and timeliness of government interventions in the form of international travel restrictions. We find that countries with higher government effectiveness and globalization are more cautious regarding the implementation of international travel restriction policies. We also find that the *de jure* economic and political dimensions and de facto social dimension of globalization have the strongest influence on the timeliness of policy implementation. We also find that countries are more likely to implement travel restrictions if their neighbor countries (in terms of share of non-resident visitor arrivals) do and that a country is over three times more likely to implement a more restrictive international travel policy measure if they have already adopted a less restrictive one first. These findings highlight the relationship between globalization and protectionist policies as governments respond to significant global events such as a public health crisis as in the case of the current COVID-19 pandemic. The findings suggest that the inclusion of such interaction variables in infectious disease models may improve the accuracy of predictions around likely time delays of disease emergence and transmission across national borders and as such, open the possibility for improved planning and coordination of transnational responses in the management of emerging and re-emerging infectious diseases into the future.

## Supplementary Information

**Additional file 1 Fig. S1.** Country-specific timeline for adoption of travel policy restrictions. Diamond markers with black outlines represent the first travel restriction implemented. Countries are ranked according to the Globalization measure. ^Countries with no travel restriction records (*n* = 32). *Countries without KOF index (*n* = 24). Five countries do not have any confirmed COVID case at time of study. **Fig. S2.** Correlation between timing of first confirmed COVID case and globalization. Pearson’s correlation (ρ) is − 0.543 (*p* < 0.001). Marker size represents the total number of COVID cases at time of data collection. Horizontal and vertical lines indicate the respective mean. **Fig. S3.** Correlations between KOF globalization index and the number of days between first COVID-19 case and travel restriction implementation (A-D) and number of COVID-19 cases at the time of first travel restriction (E-H). For each country, we calculate the measure of interest by taking the earliest of either the implementation date of the focal policy (e.g., quarantine) or the date of a more restrictive travel policy being adopted. Thus, the measures can be interpreted as the number of days lapsed since the first confirmed COVID-19 case or the number of COVID-19 cases when a ‘at-least-as-strict’ travel policy *x* was in place, respectively. Marker size represents the total number of COVID-19 cases at time of the respective policy implementation. Color indicates geographical regions (see Fig. S2 legend). Pearson’s correlations: A (*ρ* = 0.35, *p* < 0.001, *n* = 170); B (*ρ* = 0.323, *p* < 0.001, *n* = 170); C (*ρ* = 0.240, *p* = 0.0017, *n* = 170); D (*ρ* = 0.287, *p* = 0.001, *n* = 170); E (*ρ* = 0.408, *p* < 0.001, *n* = 173); F (*ρ* = 0.494, *p* < 0.001, *n* = 173); G (*ρ* = 0.502, *p* < 0.001, *n* = 173); H (*ρ* = 0.506, *p* < 0.001, *n* = 173). **Fig. S4.** Robustness checks with alternative measure of country closeness. HRs of diffusion of travel restrictions (left) and prevalence of COVID-19 in neighboring countries (right) on adoption of travel restrictions. Cap represents 95% confidence intervals. **Fig. S5.** HRs of interaction terms between globalization index and government effectiveness on adoption of travel restrictions. Cap represents 95% confidence intervals. **Fig. S6.** Estimates of the HRs of different dimensions of the globalization index on adoption of travel restrictions. Circle markers represent estimates from the main effects model (i.e., without interaction terms), with KOF indices included in the model one at the time. Triangle markers show the estimated HRs of the three KOF dimensions added together in the same model (competing effects). Diamonds show the HR estimates of the globalization dimensions in the interaction model. Cap represents 95% confidence intervals. **Table S1**. List of countries with no OxCGRT data (as of 23 September 2020). **Table S2**. List of countries with no KOF measures. **Table S3**. Placebo analysis with domestic COVID-19 responses. **Table S4**. Placebo analysis with specific domestic COVID-19 NPIs. **Table S5.** Prediction of number of COVID-19 cases at the adoption of travel restriction. **Table S6.** Prediction of COVID-19 case per capita at the adoption of travel restriction

## Data Availability

Data and materials used in the study are available online on Open Science Framework (Center for Open Science; see https://osf.io/qg6kc).

## Footnotes

1 While we follow the definition in [44], we acknowledge that there could be potential measurement errors with how the variable is measured. For example, countries may have different criteria for screening and arrival ban policies, which may vary due to the relationship with the target countries, or border closure due to non-COVID 19 reasons (e.g., war). Within country difference in levels of enforcement and coverage (e.g., state varying or selected airport screening) of the travel restrictions may also contribute to the measurement error. In addition, the measure records policy for foreign travelers and not citizens (e.g., travelling to the target country). For detailed interpretation of the variable, see https://github.com/OxCGRT/covid-policy-tracker/blob/master/documentation/interpretation_guide.md.

2 https://kof.ethz.ch/en/forecasts-and-indicators/indicators/kof-globalisation-index.html

3 See https://ethz.ch/content/dam/ethz/special-interest/dual/kof-dam/documents/Globalization/2019/KOFGI_2019_method.pdf for detailed methods on the calculation of the weights of each component and the overall index.

4 See also https://ethz.ch/content/dam/ethz/special-interest/dual/kof-dam/documents/Globalization/2019/KOFGI_2019_variables.pdf for a detailed description of each variable used in the index.

5 The average globalization index among countries (for those with KOF data, mean = 55) without OxCGRT records is slightly less than the global average (mean = 62, 95%CI = [60.1, 64]).

6 Specifically, the strength of travel restrictions, for a given country *i*, that is influenced by the country’s neighbors indexed by *j,* can be written as: $Restrictio{n}_{it}=\sum \limits_{j=1}^N{\gamma}_j Restrictio{n}_{jt}$, where 0 < *γ*_*j*_ < 1 is the share of country *i*’s visitors that come from country *j*.

7 Additionally, we check the robustness of our results using the number of physicians per 1000 people and nurses and midwives per 1000 people; we present those results in the supplementary information.

8 Regions are defined as Africa, Asia, Central America, Europe, North America, Oceania, and South America.

9 While the marginal risk set model treats each failure event as an independent process, the hazards of implementing more restrictive travel policies may not be unconditional to the occurrence of less restrictive policy being implemented. We capture this uncertainty by incorporating a time-varying variable indicating whether the country has implemented a less restrictive policy in our model.

10 Cronert [36] stratified countries by the date of the first confirmed case, however, we believe this might cause over stratification. We also group together countries that did not record their first confirmed case before April (*n* = 10).

11 This approach is also used by [36, 50] when examining the adoption rate of domestic NPI policies [38]. defines the beginning time-at-risk as the date of the first confirmed COVID-19 case of the country, thus treating countries which implemented a policy before having the first confirmed case as left censored observations. While this approach is more sensible when examining the adoption rate of domestic NPI policies (i.e., country is not yet at risk for the failure – policy implementation), it risks removing countries that engaged in precautionary strategy, i.e., implementing travel restrictions before domestic outbreaks of COVID-19.

12 Since the effect of travel restrictions might delay an outbreak of the virus, which itself might be more salient for more globalized countries, we check the correlation by censoring negative gaps (travel policy implementation before first confirmed COVID-19 case) to zero. The correlation is highly statistically significant, while the effect size is smaller (*ρ* = 0.248; *p* = 0.0011; *n* = 170). Four countries were excluded from the calculation as they have zero COVID-19 cases during the entire sample period. The correlation increases to *ρ* = 0.366 (*p* < 0.001) when the end of the sample period date is used to calculate the first policy implementation-first case gap for these countries.

13 We obtain very similar results when confirmed cases are adjusted for population size, i.e., log confirmed COVID-19 cases per million people (*ρ* = 0.397; *p* < 0.001; *n* = 173).

14 In a separate model, we control for death rate instead of number of new confirmed cases in the last seven days; the effect of either variable is statistically insignificant when added separately in the model or together.

15 The results are highly robust when we substitute other measures of democracy for electoral democracy, such as the Boix-Miller-Rosato (BMR) dichotomous coding of democracy [54], (revised) polity score and institutionalized democracy score from Polity V.

16 In addition, the effect is more pronounced if health capacity is measured with number of physicians per 1000 people. However, using the number of nurses and midwives per 1000 has no effect.

17 The HR estimates of each globalization dimension are also presented in Figure S6 (diamonds) for reference.

18 In a more sophisticated model where we include all interaction terms between each KOF subdimension (three de facto and three *de jure* dimensions) and government effectiveness, we find that the estimate of the interaction effect with *de jure* political dimension is most economically and statistically significant.

19 See https://www.who.int/nmh/resource_centre/strategic_objective6/en/

20 See https://www.weforum.org/agenda/2020/09/global-cooperation-international-united-nations-covid-19-climate-change/

21 See https://news.un.org/en/story/2020/08/1069702

22 In unreported analysis, we included policy recommendation of closure and containment. This does not alter the findings.

23 For example, we find some evidence of policy diffusion beyond the OECD context [38], while timing of domestic NPIs adoption is not sensitive to foreign COVID-19 case. We also find robust evidence that countries with a large state capacity delay implementation of domestic COVID-19 policies [36, 39]. Interestingly, we also find that countries with relevant past experience (SARS and MERS) intervened relatively early [48].

24 Notably, the HRs for gathering and internal movement restrictions are statistically (at 10% level) *larger* than one.

25 Where the former are purposed to prevent and control mass transmission of the virus within the country and the latter aims to avoid the virus from coming in to the country. In particular, countries adopt travel restrictions at an earlier stage compared to domestic policies (between mid-March to April).

26 If the country did not adopt the travel restriction, we take the COVID-19 case statistics at the end of the sample period (*n =* 4 for entry ban and *n* = 37 for total border closure). Since we use cumulative case statistics, the resulting coefficients are likely to be underestimated. This is because the sample of countries that did not implement travel bans has a higher level of globalisation than the mean, including the UK and the USA.

27 This measure captures the adoption of seven domestic containment policies and public information campaign, as well as the implementation of less restrictive travel restrictions. See https://github.com/OxCGRT/covid-policy-tracker/blob/master/documentation/index_methodology.md for the construction of the stringency index.
