# Supplementary material for: How does globalization affect COVID-19 responses?
Source: Global Health. 2021 May 20;17:57. doi: 10.1186/s12992-021-00677-5 (PMC8134968; doi:10.1186/s12992-021-00677-5)
Supplement: Supplementary file 1 — Additional file 1 Fig. S1. Country-specific timeline for adoption of travel policy restrictions. Diamond markers with black outlines represent the first travel restriction implemented. Countries are ranked according to the Globalization measure. ^Countries with no travel restriction records (n = 32). *Countries without KOF index (n = 24). Five countries do not have any confirmed COVID case at time of study. Fig. S2. Correlation between timing of first confirmed COVID case and globalization. Pearson’s correlation (ρ) is − 0.543 (p < 0.001). Marker size represents the total number of COVID cases at time of data collection. Horizontal and vertical lines indicate the respective mean. Fig. S3. Correlations between KOF globalization index and the number of days between first COVID-19 case and travel restriction implementation (A-D) and number of COVID-19 cases at the time of first travel restriction (E-H). For each country, we calculate the measure of interest by taking the earliest of either the implementation date of the focal policy (e.g., quarantine) or the date of a more restrictive travel policy being adopted. Thus, the measures can be interpreted as the number of days lapsed since the first confirmed COVID-19 case or the number of COVID-19 cases when a ‘at-least-as-strict’ travel policy x was in place, respectively. Marker size represents the total number of COVID-19 cases at time of the respective policy implementation. Color indicates geographical regions (see Fig. S2 legend). Pearson’s correlations: A (ρ = 0.35, p < 0.001, n = 170); B (ρ = 0.323, p < 0.001, n = 170); C (ρ = 0.240, p = 0.0017, n = 170); D (ρ = 0.287, p = 0.001, n = 170); E (ρ = 0.408, p < 0.001, n = 173); F (ρ = 0.494, p < 0.001, n = 173); G (ρ = 0.502, p < 0.001, n = 173); H (ρ = 0.506, p < 0.001, n = 173). Fig. S4. Robustness checks with alternative measure of country closeness. HRs of diffusion of travel restrictions (left) and prevalence of COVID-19 in neighboring countries (right) on adoptio [file 12992_2021_677_MOESM1_ESM.docx]

**Supplementary information for**

**How Does Globalization Affect COVID-19 Responses?**

Steve J. Bickley^1,2^, Ho Fai Chan^1,2,*^, Ahmed Skali^3^, David Stadelmann^2,4,5^ and Benno Torgler^1,2,5^

Corresponding author. E-mail address: [hofai.chan@qut.edu.au](mailto:hofai.chan@qut.edu.au)

^1^ School of Economics and Finance, Queensland University of Technology, 2 George St, Brisbane QLD 4000, Australia

^2^ Centre for Behavioural Economics, Society and Technology (BEST), 2 George St, Brisbane QLD 4000, Australia

^3^ Deakin University, Victoria, Australia.

^4^ University of Bayreuth, Bayreuth, Germany.

^5^ CREMA – Centre for Research in Economics, Management, and the Arts, Südstrasse 11, CH-8008 Zürich, Switzerland.

Sensitivity tests on Alternative proximity dimensions.

We check our results with travel restriction policy adoption (and cumulative COVID-19 cases) of neighboring countries derived from other dimensions of country proximity; i.e., 1) share of total gross bilateral export or import [1], 2) geographical distance (CEPII’s GeoDist database [2]^^[[1]](#footnote-1)^^, 3) absolute psychic distance [3]^^[[2]](#footnote-2)^^, and 4) cross-national distance from Wharton Management (the updated data of [4])^^[[3]](#footnote-3)^^.

The data used to construct economic proximity are based on the share of total gross bilateral exports or imports in 2018, broken down by country, which was obtained from the World Integrated Trade Solution – World Bank under the UN COMTRADE Standard International Trade Classification, Revision 4 (SITC Rev4) 2018 [1]. Bilateral geographical proximity is calculated using the great circle formula between the most populated cities and the capital. Population weighted distance produces very similar results (unreported). Psychic distance includes closeness in education, religion, education, democracy, and industrial development dimensions (see [3] for details). Social (difference in political ideology) and language (linguistic) dimensions are omitted due to insufficient observations and low variability in the measure. Lastly, we consider six distance dimensions, i.e., demographic, economic, geographic, knowledge, and political, from [4]. Culture and finance dimensions were omitted due to small country samples. Mahalanobis distances were calculated based on the latest yearly data. For example, economic distance was based on income, inflation, import, and export data from the WDI in 2016. As described in the main text, we use the share of inbound arrivals or export and import as the weights to construct the variables for our analysis; for all other distances, we use the inverse of the raw scores.

In Figure S4, we show the estimates of the hazard ratios for the variables *neighbor restriction adoption* and *neighbor COVID-19 case* calculated using different distance measures to define neighboring countries. As expected, countries are most responsive to policy adoption and number of recent confirmed COVID-19 cases of the countries with the largest share of tourist arrivals. In other words, variables constructed using inbound tourism have the highest predictability on the adoption of international travel restrictions. Among other country proximities, geographic distance and global connectedness distance are also able to explain the timing of travel policy implementation. The HR of the main (interaction) effects in each model are shown in Figure S5. While the mean hazard ratios are consistently estimated between 0.7 to 0.9, they are less precisely estimated for models that use psychic distances.

**
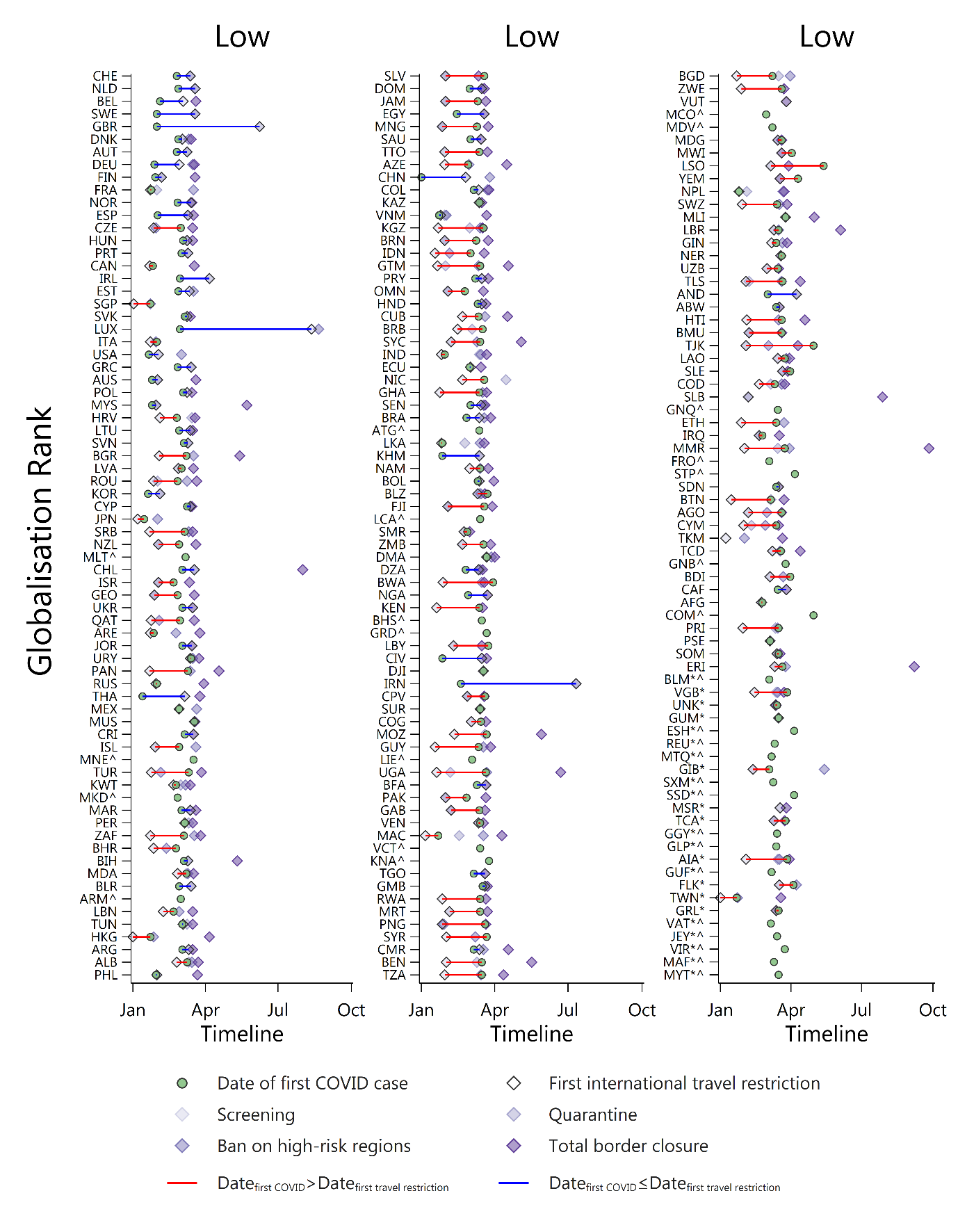
**

**Fig. S1.** Country-specific timeline for adoption of travel policy restrictions. Diamond markers with black outlines represent the first travel restriction implemented. Countries are ranked according to the Globalization measure. ^Countries with no travel restriction records (*n*=32). *Countries without KOF index (*n*=24). Five countries do not have any confirmed COVID case at time of study.

**
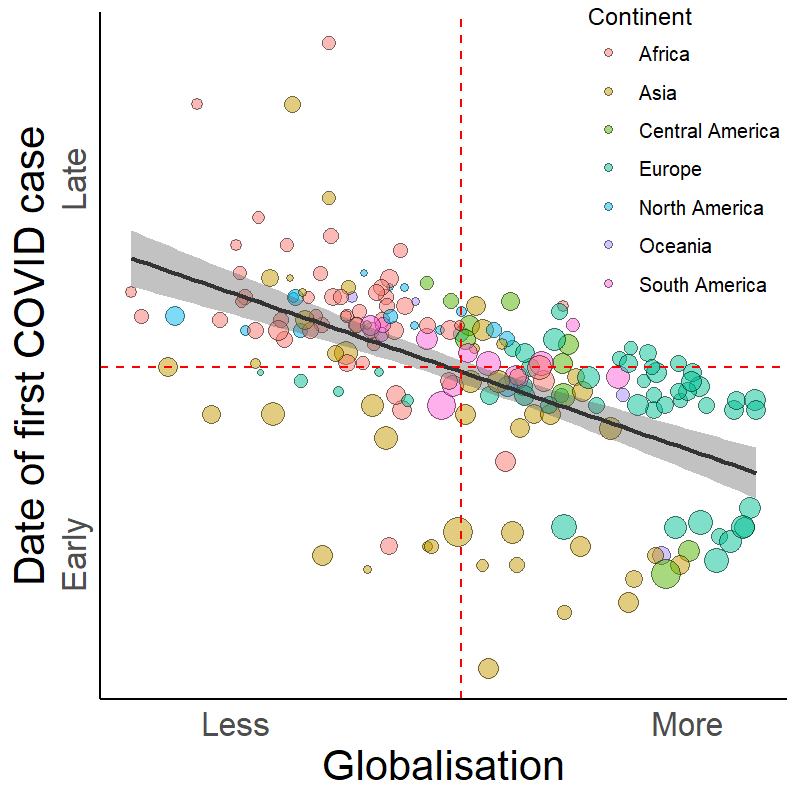
**

**Fig. S2.** Correlation between timing of first confirmed COVID case and globalization. Pearson’s correlation (ρ) is -0.543 (*p*<0.001). Marker size represents the total number of COVID cases at time of data collection. Horizontal and vertical lines indicate the respective mean.


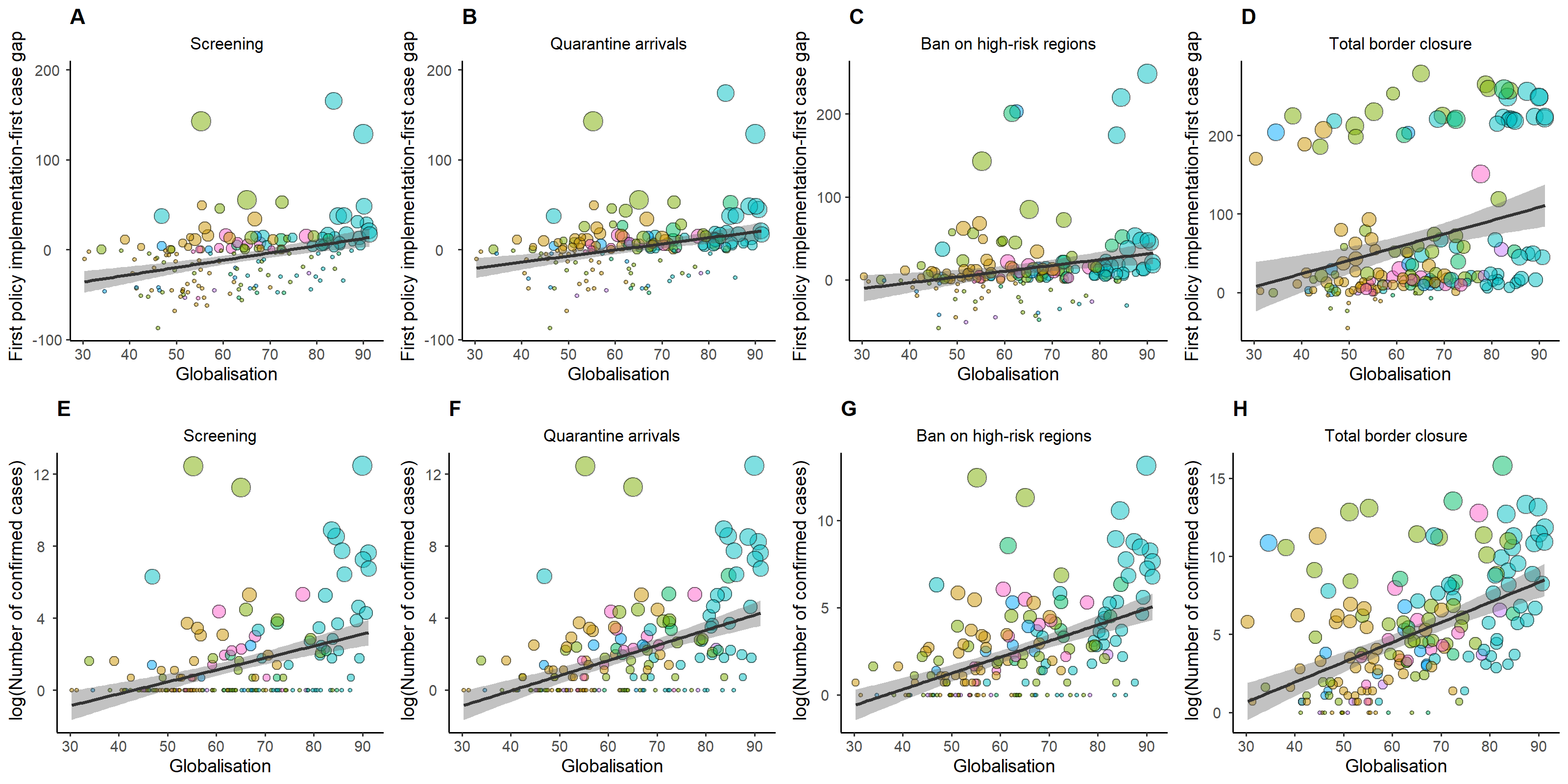


**Fig. S3.** Correlations between KOF globalization index and the number of days between first COVID-19 case and travel restriction implementation (**A-D**) and number of COVID-19 cases at the time of first travel restriction (**E-H**). For each country, we calculate the measure of interest by taking the earliest of either the implementation date of the focal policy (e.g., quarantine) or the date of a more restrictive travel policy being adopted. Thus, the measures can be interpreted as the number of days lapsed since the first confirmed COVID-19 case or the number of COVID-19 cases when a ‘at-least-as-strict’ travel policy *x* was in place, respectively. Marker size represents the total number of COVID-19 cases at time of the respective policy implementation. Color indicates geographical regions (see Fig. S2 legend). Pearson’s correlations: **A** (*ρ*=0.35, *p*<0.001, *n*=170); **B** (*ρ*=0.323, *p*<0.001, *n*=170); **C** (*ρ*=0.240, *p*=0.0017, *n*=170); **D** (*ρ*=0.287, *p*=0.001, *n*=170); **E** (*ρ*=0.408, *p*<0.001, *n*=173); **F** (*ρ*=0.494, *p*<0.001, *n*=173); **G** (*ρ*=0.502, *p*<0.001, *n*=173); **H** (*ρ*=0.506, *p*<0.001, *n*=173).


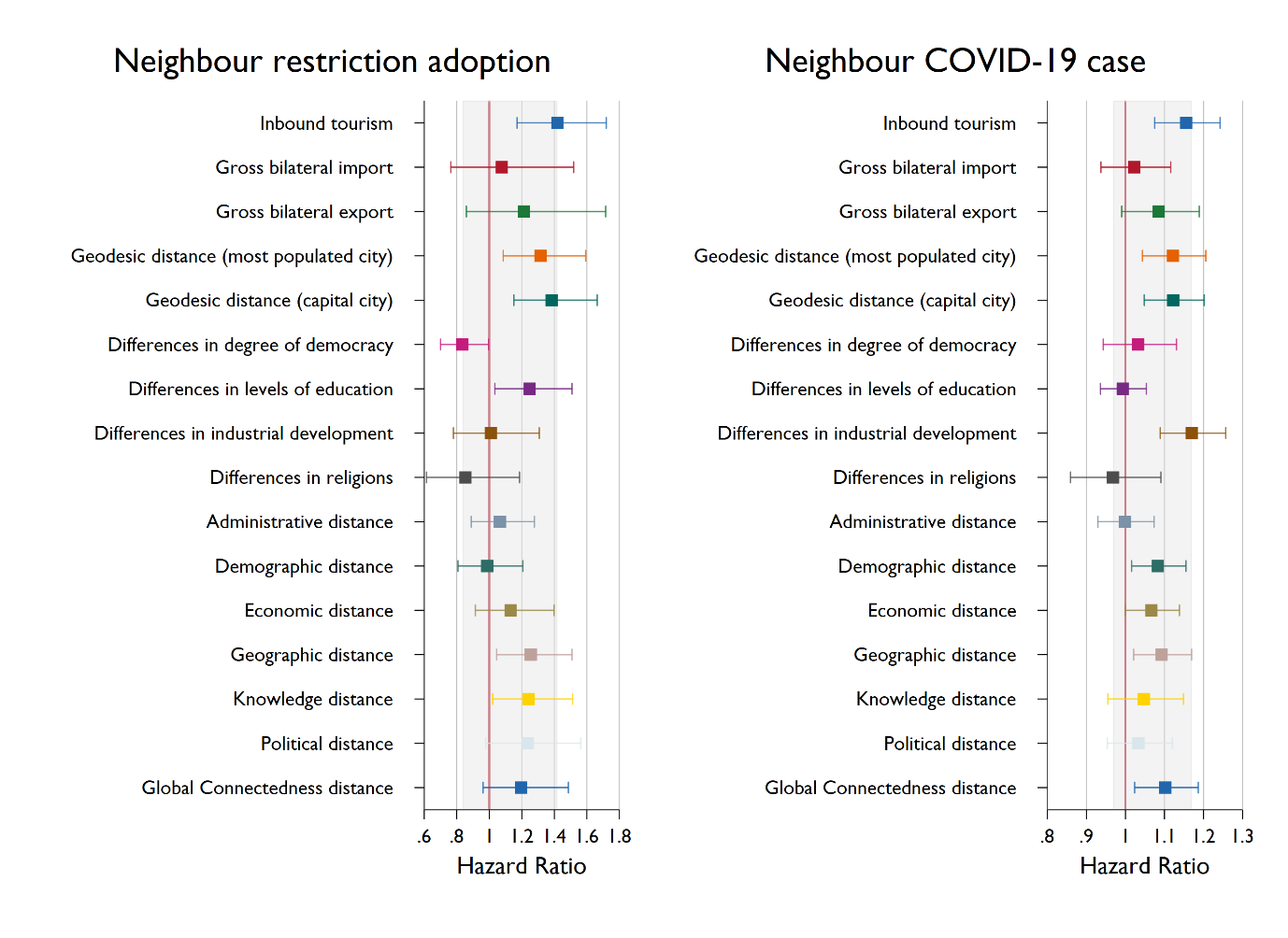


**Fig. S4.** Robustness checks with alternative measure of country closeness. HRs of diffusion of travel restrictions (left) and prevalence of COVID-19 in neighboring countries (right) on adoption of travel restrictions. Cap represents 95% confidence intervals.


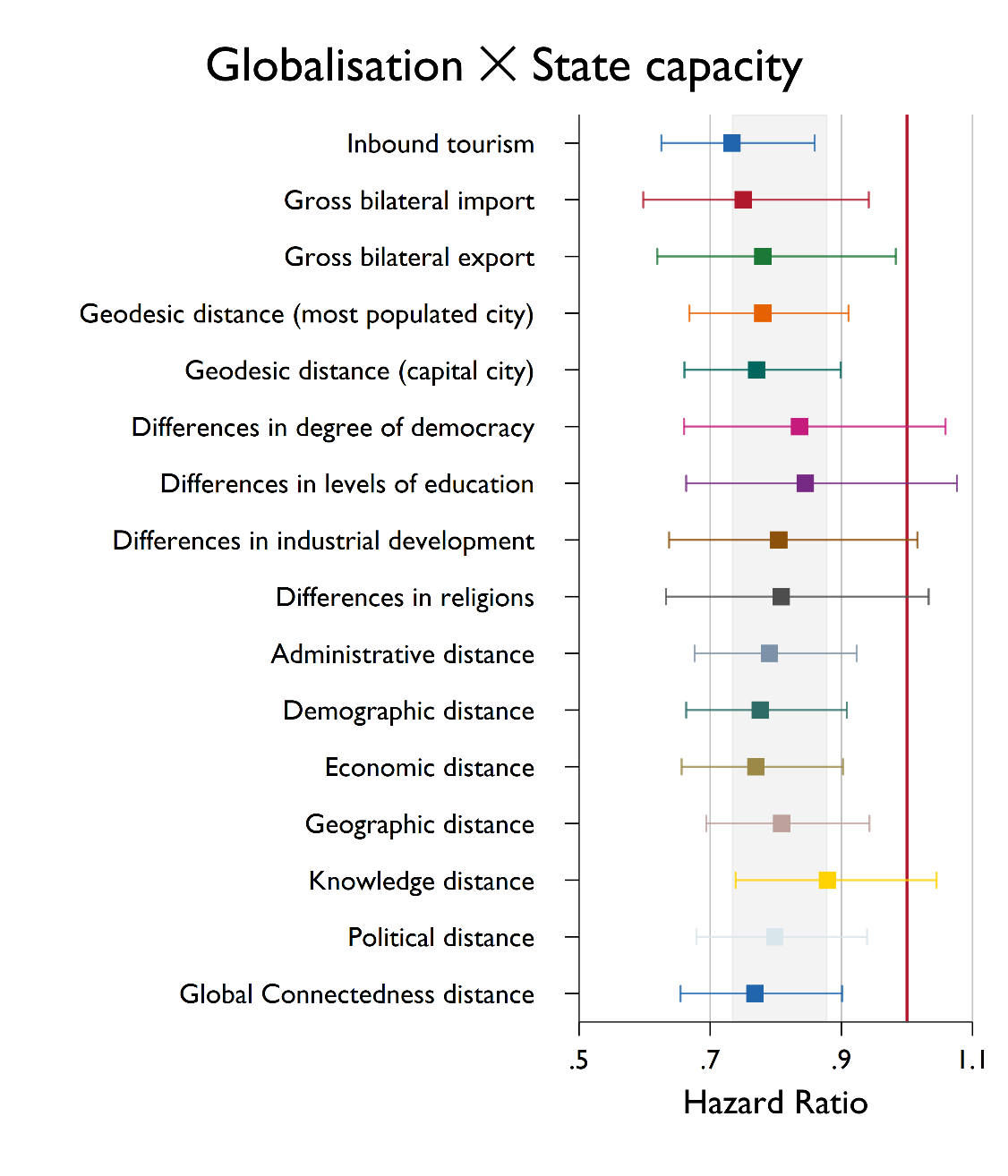


**Fig. S5.** HRs of interaction terms between globalization index and government effectiveness on adoption of travel restrictions. Cap represents 95% confidence intervals.


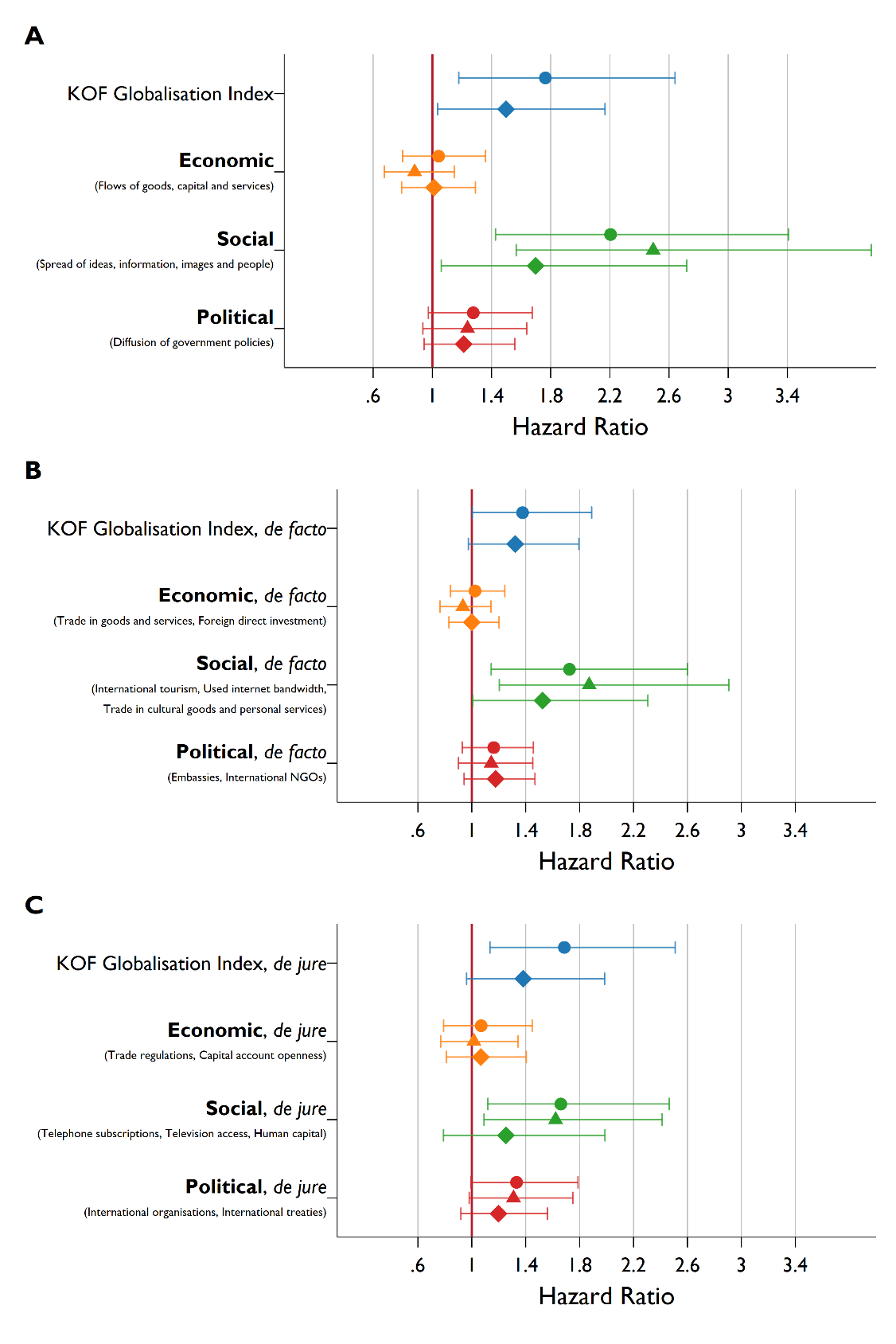


**Fig. S6.** Estimates of the HRs of different dimensions of the globalization index on adoption of travel restrictions. Circle markers represent estimates from the main effects model (i.e., without interaction terms), with KOF indices included in the model one at the time. Triangle markers show the estimated HRs of the three KOF dimensions added together in the same model (competing effects). Diamonds show the HR estimates of the globalization dimensions in the interaction model. Cap represents 95% confidence intervals.

**Table S1**. List of countries with no OxCGRT data (as of 23 September 2020)

| **Country** | **KOF** |
| --- | --- |
| Antigua and Barbuda | 60.50 |
| Armenia | 68.60 |
| Comoros | 36.81 |
| Equatorial Guinea | 44.96 |
| Faeroe Islands | 42.92 |
| French Guiana |  |
| Grenada | 55.54 |
| Guadeloupe |  |
| Guernsey |  |
| Guinea-Bissau | 40.93 |
| Jersey |  |
| Liechtenstein | 54.56 |
| Maldives | 50.24 |
| Malta | 77.87 |
| Martinique |  |
| Mayotte |  |
| Monaco | 50.53 |
| Montenegro | 72.10 |
| North Macedonia | 71.10 |
| Réunion |  |
| Saint Barthélemy |  |
| Saint Kitts and Nevis | 53.05 |
| Saint Lucia | 57.91 |
| Saint Martin (French part) |  |
| Saint Vincent and the Grenadines | 53.37 |
| Sao Tome and Principe | 42.67 |
| Sint Maarten (Dutch part) |  |
| The Bahamas | 55.61 |
| Vatican |  |
| Western Sahara |  |

**Table S2**. List of countries with no KOF measures

| **Country** |
| --- |
| Anguilla |
| British Virgin Islands |
| Falkland Islands |
| Gibraltar |
| Greenland |
| Guam |
| Kosovo |
| Montserrat |
| South Sudan |
| Taiwan |
| Turks and Caicos Islands |

**Table S3**. Placebo analysis with domestic COVID-19 responses.

|  |  |  |  |  |  |  |
| --- | --- | --- | --- | --- | --- | --- |
| KOF Globalization Index | 1.20^**^ | 1.22^*^ | 2.42^***^ | 2.44^***^ |  |  |
|  | (0.0769) | (0.107) | (0.506) | (0.530) |  |  |
| KOF*Government Effectiveness |  |  |  | 1.01 |  |  |
|  |  |  |  | (0.0655) |  |  |
| Neighbor NPIs adoption |  | 1.27^**^ | 1.19^*^ | 1.19^*^ |  |  |
|  |  | (0.0946) | (0.104) | (0.105) |  |  |
| Neighbor COVID-19 case (7-day total, log) |  | 1.00 | 1.03 | 1.03 |  |  |
|  |  | (0.0319) | (0.0459) | (0.0458) |  |  |
| Domestic COVID-19 case (7-day total, log) |  | 0.96 | 0.91^†^ | 0.91^†^ |  |  |
|  |  | (0.0420) | (0.0463) | (0.0462) |  |  |
| Weekends |  | 0.38^**^ | 0.61 | 0.61 |  |  |
|  |  | (0.122) | (0.187) | (0.187) |  |  |
| Government Effectiveness (WGI) |  |  | 0.60^**^ | 0.60^**^ |  |  |
|  |  |  | (0.0968) | (0.0986) |  |  |
| Electoral democracy index |  |  | 1.15 | 1.15 |  |  |
|  |  |  | (0.127) | (0.128) |  |  |
| GDP per capita (log) |  |  | 1.19^†^ | 1.19^†^ |  |  |
|  |  |  | (0.125) | (0.124) |  |  |
| Unemployment (%) |  |  | 1.01 | 1.01 |  |  |
|  |  |  | (0.0158) | (0.0160) |  |  |
| GINI index |  |  | 1.00 | 1.00 |  |  |
|  |  |  | (0.0118) | (0.0118) |  |  |
| Hospital beds (per 1k people) |  |  | 0.98 | 0.98 |  |  |
|  |  |  | (0.0379) | (0.0394) |  |  |
| Population ages 65+ (%) |  |  | 0.93^**^ | 0.93^**^ |  |  |
|  |  |  | (0.0232) | (0.0235) |  |  |
| Urban population (%) |  |  | 0.99^*^ | 0.99^*^ |  |  |
|  |  |  | (0.00471) | (0.00476) |  |  |
| Population density (log) |  |  | 1.11^*^ | 1.11^*^ |  |  |
|  |  |  | (0.0457) | (0.0456) |  |  |
| MERS or SARS experience |  |  | 2.08^*^ | 2.08^*^ |  |  |
|  |  |  | (0.627) | (0.626) |  |  |
| Continent |  |  |  |  |  |  |
| Africa |  |  | 0.50^*^ | 0.50^*^ |  |  |
|  |  |  | (0.165) | (0.166) |  |  |
| Asia |  |  | 0.84 | 0.84 |  |  |
|  |  |  | (0.272) | (0.273) |  |  |
| Europe |  |  | (ref.) | (ref.) |  |  |
| Central America |  |  | 0.42^*^ | 0.43^*^ |  |  |
|  |  |  | (0.178) | (0.183) |  |  |
| North America |  |  | 0.59^†^ | 0.59^†^ |  |  |
|  |  |  | (0.170) | (0.170) |  |  |
| Oceania |  |  | 0.72 | 0.73 |  |  |
|  |  |  | (0.389) | (0.400) |  |  |
| South America |  |  | 1.32 | 1.33 |  |  |
|  |  |  | (0.476) | (0.486) |  |  |
| Num. obs. | 320402 | 274632 | 218324 | 218324 |  |  |
| Num. countries | 173 | 158 | 121 | 121 |  |  |
| Num. failures | 1300 | 1202 | 871 | 871 |  |  |
| Pseudo R^2^ | 0.003 | 0.008 | 0.030 | 0.030 |  |  |
| Log likelihood | -4760.181 | -4238.712 | -2791.702 | -2791.685 |  |  |

Notes: Hazard ratios. Standard errors (clustered at country level) in parentheses. † *p* < .10; * *p* < .05; ** *p* < .01; *** *p* < .001.

**Table S4**. Placebo analysis with specific domestic COVID-19 NPIs.

|  | School closure | Workplace closure | Public events cancellation | Gathering restrictions | Public transport closure | Stay at home requirements | Internal mobility restrictions |
| --- | --- | --- | --- | --- | --- | --- | --- |
| KOF | 2.27^*^ | 2.98^*^ | 2.95^*^ | 2.84^**^ | 2.58 | 4.94^**^ | 5.33^**^ |
|  | (0.887) | (1.295) | (1.413) | (0.953) | (2.142) | (2.721) | (3.328) |
| Government Effectiveness | 0.72 | 0.60 | 0.51 | 0.50^*^ | 0.60 | 0.32^*^ | 0.18^***^ |
|  | (0.272) | (0.208) | (0.212) | (0.137) | (0.367) | (0.142) | (0.0784) |
| KOF* Government Effectiveness | 0.89 | 1.14 | 0.91 | 1.19^†^ | 0.74 | 0.86 | 1.39^†^ |
|  | (0.116) | (0.143) | (0.135) | (0.124) | (0.235) | (0.202) | (0.238) |
| Controls | YES | YES | YES | YES | YES | YES | YES |
| Num. obs. | 26313 | 34256 | 13620 | 47074 | 26307 | 49000 | 21754 |
| Num. countries | 121 | 121 | 121 | 121 | 121 | 121 | 121 |
| Num. failures | 191 | 156 | 93 | 260 | 32 | 83 | 56 |
| Pseudo R^2^ | 0.062 | 0.050 | 0.046 | 0.053 | 0.082 | 0.132 | 0.092 |
| Log likelihood | -569.0 | -486.6 | -284.9 | -799.1 | -111.3 | -255.3 | -182.0 |

Notes: Hazard ratios. Standard errors (clustered at country level) in parentheses. † *p* < .10; * *p* < .05; ** *p* < .01; *** *p* < .001.

**Table S5.** Prediction of number of COVID-19 cases at the adoption of travel restriction.

|  | Total confirmed COVID-19 cases (log) | | | |
| --- | --- | --- | --- | --- |
|  | Screening | Quarantine | Ban high-risk | Total lockdown |
| KOF Globalization Index | 0.78^†^ | 1.00^*^ | 0.68 | -0.41 |
|  | (0.410) | (0.421) | (0.439) | (0.524) |
| Date first case | 0.016^*^ | 0.0066 | 0.0062 | -0.037^**^ |
|  | (0.00697) | (0.0103) | (0.0133) | (0.0114) |
| Government Effectiveness | 0.16 | -0.089 | 0.30 | 0.43 |
|  | (0.363) | (0.446) | (0.500) | (0.462) |
| Electoral democracy index | -0.53^*^ | -0.56^*^ | -0.64^*^ | -1.20^***^ |
|  | (0.240) | (0.225) | (0.281) | (0.321) |
| GDP per capita (log) | 0.56^*^ | 0.52^†^ | 0.27 | 0.48 |
|  | (0.249) | (0.276) | (0.336) | (0.327) |
| Unemployment, total (%) | 0.013 | -0.014 | -0.017 | -0.082^*^ |
|  | (0.0315) | (0.0351) | (0.0376) | (0.0359) |
| GINI index | -0.040 | -0.067^*^ | -0.051 | -0.023 |
|  | (0.0279) | (0.0303) | (0.0332) | (0.0326) |
| Hospital beds (per 1,000 people) | -0.12 | -0.12 | -0.16^†^ | -0.074 |
|  | (0.111) | (0.101) | (0.0955) | (0.123) |
| Population ages 65+ (%) | 0.056 | 0.080 | 0.13^*^ | 0.14^*^ |
|  | (0.0591) | (0.0601) | (0.0638) | (0.0633) |
| Urban population (%) | -0.019^†^ | -0.0052 | 0.0098 | 0.025^†^ |
|  | (0.0110) | (0.0122) | (0.0134) | (0.0142) |
| Population density (log) | 0.070 | 0.15 | 0.069 | 0.36^†^ |
|  | (0.141) | (0.153) | (0.146) | (0.183) |
| MERS or SARS experience | -0.95 | 0.14 | -0.18 | -2.72^*^ |
|  | (0.947) | (0.768) | (0.819) | (1.083) |
| Average stringency index | 0.26^***^ | 0.25^***^ | 0.19^***^ | 0.15^***^ |
|  | (0.0251) | (0.0297) | (0.0219) | (0.00986) |
| Constant | -357.9^*^ | -146.9 | -138.6 | 813.7^**^ |
|  | (154.1) | (227.7) | (292.0) | (250.6) |
| Continent dummies | Yes | Yes | Yes | Yes |
| Number of countries | 118 | 118 | 118 | 118 |
| Prob. > *F* | 0.000 | 0.000 | 0.000 | 0.000 |
| *R^2^* | 0.749 | 0.703 | 0.697 | 0.826 |

Notes: OLS estimates correspond to Figure 6A with control variables. Standard errors (heteroskedasticity-robust) in parentheses. † *p* < .10; * *p* < .05; ** *p* < .01; *** *p* < .001.

**Table S6.** Prediction of COVID-19 case per capita at the adoption of travel restriction.

|  | COVID-19 case per capita (log) | | | |
| --- | --- | --- | --- | --- |
|  | Screening | Quarantine | Ban high-risk | Total lockdown |
| KOF Globalization Index | 1.79^*^ | 1.75^*^ | 0.85 | -1.10 |
|  | (0.698) | (0.860) | (0.778) | (0.674) |
| Date first case | 0.083^***^ | 0.080^***^ | 0.083^***^ | -0.030^*^ |
|  | (0.0142) | (0.0184) | (0.0205) | (0.0128) |
| Government Effectiveness | 0.71 | 0.34 | 0.58 | 0.48 |
|  | (0.733) | (0.858) | (0.870) | (0.658) |
| Electoral democracy index | -0.77^†^ | -1.01^**^ | -0.93^*^ | -1.07^*^ |
|  | (0.418) | (0.369) | (0.441) | (0.421) |
| GDP per capita (log) | 0.93^*^ | 1.23^*^ | 0.92 | 1.11^*^ |
|  | (0.434) | (0.496) | (0.582) | (0.431) |
| Unemployment, total (%) | 0.030 | 0.028 | -0.055 | -0.10^†^ |
|  | (0.0537) | (0.0617) | (0.0695) | (0.0559) |
| GINI index | -0.075 | -0.12^*^ | -0.057 | -0.038 |
|  | (0.0469) | (0.0526) | (0.0590) | (0.0494) |
| Hospital beds (per 1,000 people) | -0.13 | -0.12 | -0.32^*^ | -0.086 |
|  | (0.180) | (0.152) | (0.148) | (0.144) |
| Population ages 65+ (%) | 0.019 | 0.069 | 0.23^*^ | 0.099 |
|  | (0.101) | (0.0973) | (0.0975) | (0.0704) |
| Urban population (%) | -0.034^†^ | -0.022 | 0.017 | 0.034^†^ |
|  | (0.0183) | (0.0203) | (0.0230) | (0.0190) |
| Population density (log) | 0.24 | 0.43^†^ | 0.050 | 0.31 |
|  | (0.230) | (0.246) | (0.250) | (0.268) |
| MERS or SARS experience | -1.86 | 0.54 | 0.90 | -3.17^*^ |
|  | (1.758) | (1.106) | (1.254) | (1.325) |
| Average stringency index | 0.30^***^ | 0.27^***^ | 0.20^***^ | 0.13^***^ |
|  | (0.0357) | (0.0418) | (0.0323) | (0.0112) |
| Constant | -1825.0^***^ | -1773.0^***^ | -1842.8^***^ | 644.2^*^ |
|  | (313.1) | (402.8) | (449.2) | (280.9) |
| Continent dummies | Yes | Yes | Yes | Yes |
| Number of countries | 118 | 118 | 118 | 118 |
| Prob. > *F* | 0.000 | 0.000 | 0.000 | 0.000 |
| *R^2^* | 0.696 | 0.649 | 0.647 | 0.752 |

Notes: OLS estimates correspond to Figure 6B with control variables. Standard errors (heteroskedasticity-robust) in parentheses. † *p* < .10; * *p* < .05; ** *p* < .01; *** *p* < .001.

**References**

1. UN COMTRADE. United Nations Commodity Trade Statistics Database. United Nations Statisitcal Division (NewYork: United Nations). 2020.
2. Mayer T, Zignago S. Notes on CEPII’s distances measures: the GeoDist database CEPII working paper 2011–25; 2011.
3. Dow D, Karunaratna A. Developing a multidimensional instrument to measure psychic distance stimuli. J Int Bus Stud. 2006;37:578–602.
4. Berry H, Guillén MF, Zhou N. An institutional approach to cross-national distance. J Int Bus Stud. 2010 Dec 1;41(9):1460–80.

1. <http://www.cepii.fr/cepii/en/bdd_modele/presentation.asp?id=6> [↑](#footnote-ref-1)
2. <http://dow.net.au/?page_id=29> [↑](#footnote-ref-2)
3. http://www.management.wharton.upenn.edu/guillen/Distance_Data_Downloads.htm [↑](#footnote-ref-3)
